# An investigation into the physico-chemical characteristics and performance of surface-treated areca fibers

**DOI:** 10.1038/s41598-025-33181-6

**Published:** 2026-01-04

**Authors:** G. Manavendra, Vinay Atgur, S. Basavarajappa, B. Nageswara Rao, N. R. Banapurmath, S. Dhanalakshmi, Ashok M. Sajjan, Irfan Anjum Badruddin, M. A. Umarfarooq, S. M. Abdul Khader, Essam R. I. Mahmoud

**Affiliations:** 1 Department of Mechanical Engineering, Bapuji Institute of Engineering and Technology (BIET), Davangere, 577055 Karnataka India; 2 Department of Mechanical Engineering, Koneru Lakshmaiah Educational Foundation, Vaddeswaram, 522502 Andhra Pradesh India; 3 Department of Mechanical Engineering, GM University, Davangere, 577006 Karnataka India; 4 Centre of Excellence in Material Science, School of Mechanical Engineering, KLE Technological University, Hubballi, 580031 India; 5 Department of Mechanical Engineering, King Khalid University, 61421 Abha, Saudi Arabia; 6 Center for Material Science, Department of Mechanical Engineering, Karpagam Academy of Higher Education, Coimbatore, Tamil Nadu India; 7 Department of Mechanical & Industrial Engineering, Manipal Institute of Technology, Manipal Academy of Higher Education, Manipal, 576104 India; 8 Department of Mechanical Engineering, Islamic University of Madinah, 41411 Madinah, Saudi Arabia

**Keywords:** Alkaline treatment, Areca fibers, Areca husk, Natural fibers, Percentage elongation, Ultimate tensile strength, Young’s modulus, Engineering, Mechanical engineering

## Abstract

This research explores chemical and mechanical treatments to enhance the properties of areca husk fibers, an abundant resource in Karnataka. A primary focus of this work is the comprehensive characterization of these fibers. Fibers, averaging 35 mm in length and 300 $\mu m$ in diameter, were extracted from the husk. Chemical analysis revealed a predominantly cellulose composition with low lignin content. Following extraction, the fibers underwent alkaline treatment, and their physical, mechanical, and chemical characteristics were thoroughly assessed. Significant improvements in mechanical properties, including tensile strength, Young’s modulus, and elongation at break, were observed after alkaline treatment. The microfibrillar angle was theoretically estimated, and the calculated theoretical strength was compared with experimental results. Morphological analysis further elucidated the fiber’s structural characteristics. Overall, areca fiber properties were found to be comparable to other natural fibers, highlighting their potential for developing eco-friendly and cost-effective composites.

## Introduction

Fibers originating from plants, such as the soap bark of *Acacia caesia*, the bark of *Grewia Monticola Sond* (GMS), and the aerial root fibers of *Ficus retusa L.*, are increasingly valued as components with high potential in advanced composite materials. These fibers are rich in cellulose, lignin, and hemicellulose, offering desirable characteristics like being lightweight, renewable, biodegradable, and possessing good thermal and mechanical properties^1–4^. Collectively, Natural Bark Fibers (NBFs) present a promising and eco-friendly alternative to synthetic fibers in various industries^5^. Cellulose Microcrystals (CMC), when derived from sources such as discarded cotton textiles or agricultural biomass, is formally categorized as a waste product. Similarly, Medium-Density Fiberboard (MDF) is designated as a waste material due to the significant quantities produced and the considerable volume of scrap generated across its life-cycle, encompassing manufacturing off-cuts, fabrication errors, and end-of-life discarded items. The primary intent behind classifying both materials as waste is to encourage their beneficial reuse and prevent their disposal in landfills. This approach facilitates their reclamation as valuable raw materials for manufacturing novel goods or for various other value-added industrial applications. Furthermore, integrating these reclaimed materials, such as CMC and MDF waste, into thermoplastic polymer matrices offers both an ingenious and economical solution. This practice not only measurably enhances the composite’s mechanical integrity and its resistance to water absorption but simultaneously yields considerable ecological advantages through concentrated efforts to minimize waste generation^6^.

### Areca fibers

Areca fibers represent a promising area in both research and applications, due to their sustainability and favorable mechanical properties. These environmentally friendly fibers are expected to gain increasing importance across diverse industries, from construction to textiles. The *Areca catechu* plant, the source of these fibers, thrives in tropical regions such as the Pacific, Eastern Africa, and Asia, with significant cultivation in Malaysia, the Philippines, India, and Sri Lanka^7,8^. According to the Food and Agriculture Organization of the United Nations^9^, India, Myanmar, Bangladesh, China, and Indonesia are among the leading global producers of betel nut. In India and Sri Lanka, *Areca catechu* trees are extensively cultivated for the traditional consumption of betel nut alongside betel leaves and limestone paste^10^. The areca tree is an upright palm, growing 10 to 20 m tall. Its trunk is straight, slender, and unbranched, with a circumference of 10 to 15 cm, featuring circular scars left by fallen leaf sheaths or fronds^11^. Large leaves, 1.5 to 2 m long, crown the tree, consisting of numerous pinnate-shaped leaflets, with 8 to 12 fronds commonly found in the upper section^12^.

**Fig. 1:**
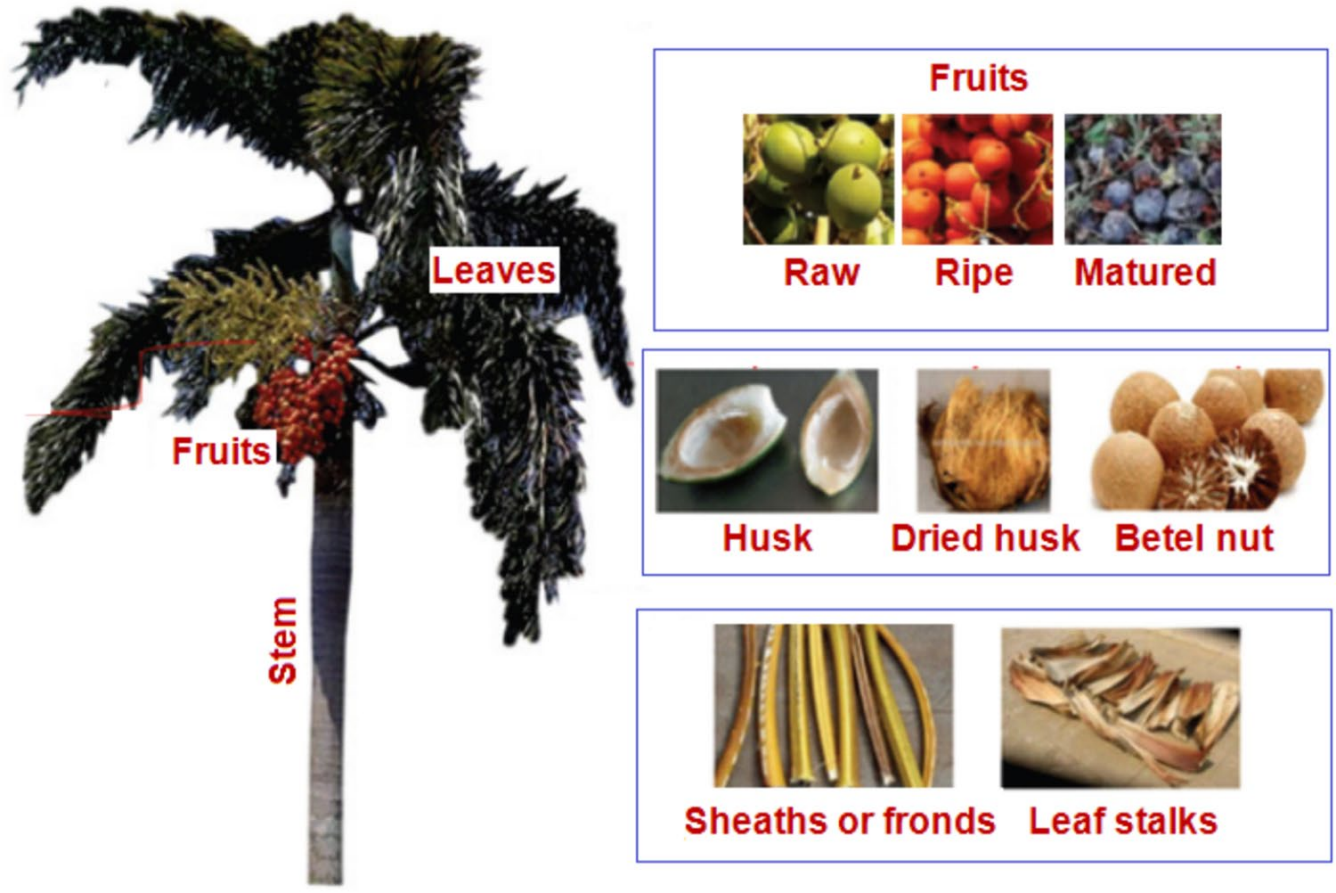
Areca catechu tree^5^.

The Areca catechu tree (Fig. 1) yields distinct types of fibers from its three main components: fruit, leaves, and stem. Betel husk fibers are derived from the fruit. Leaf fibers originate from two distinct areas: the leaf stalks and the fronds or sheaths. To allow for detailed examination of fiber distribution along its length and diameter, stem fibers are categorized into top, middle, and bottom segments. Areca fibers, available as short and long strands of varying diameters, and woven mats made from them, are suitable for composite reinforcement. Notably, areca sheath or frond fibers offer superior tensile strength and lower density compared to coconut and palm fibers^13^, attributed to their high cellulose content and considerable length.

### Literature survey

To enhance their utility in diverse applications, areca fibers—extracted from various parts of the areca palm—undergo chemical modification. Treatments often involve substances like sodium bicarbonate, potassium permanganate, chromium sulfate, sodium hydroxide, acrylic acid, alkylation, and acrylation, which commonly result in reduced fiber diameter and increased density^14–17^. A specific improvement comes from benzoyl chloride treatments on areca sheath and frond fibers, which significantly boost their tensile and flexural strength^18,19^. These fibers intrinsically exhibit a high cellulose content (57.35%–65.02%), densities from 0.48 to 1.34 g/cm^3^, and tensile strengths of 159.62–231.66 MPa. Additionally, they boast a low water absorption capacity, distinguishing them from many other fiber types^20–22^. They produce panel boards, decking, railing, and lightweight structural bearing sheets^19,22^.

These robust characteristics make areca fibers excellent reinforcement in bio-reinforced polymer composites for automotive and structural sectors^23–32^. They are effectively incorporated into epoxy composites, where adding co-fillers like silicon carbide and increasing filler content enhances mechanical properties^33^. Furthermore, areca fiber- natural latex composites with silane coupling agents like Si-69 improved tensile and tear strength^34^. Surface-modified areca fibers have optimized the tensile strength and Young’s modulus of LLDPE composites at a 5% reinforcement level^35^. Similarly, Areca Palm Leaf Stalk Fibers (APLSF) treated with 5% alkali exhibit superior tensile strength, modulus, and percentage elongation, affirming their broad utility in composite design^36^. The pulp from areca nuts having shorter fiber lengths and higher acid-insoluble lignin content, is well-suited for manufacturing packaging paper^37^. Randomly oriented alkali-treated areca fibers strengthening silty sand soil mixtures aid in construction projects and soil stabilization^38^.

### Research in India

As the world’s largest areca nut producer, India, particularly Karnataka’s key districts like Shivamogga, Chikkamagaluru, and Uttara Kannada, generates a substantial volume of areca husk waste^38^. Currently, this fiber-rich biomass is largely underutilized, often discarded or burned; burning releases carbon-rich particulates, degrading air quality, while general underutilization creates significant environmental burdens and economic losses.

Investigating areca fibers in India therefore represents a strategic step towards sustainable development^39,40^. Converting this waste into value-added materials—such as paper, bio-composites, textiles, and carbon adsorbents—offers a promising pathway to reduce waste and pollution.

Leading institutions are actively engaged in research, focusing on areca fiber extraction, characterization, and the impact of cellulose extraction on composite properties^41^. Areca fibers contain ~ 63% cellulose, 0.34% wax, and density ~ 1.34 g/cc, making them strong candidates for composites^42^. Further studies involve the development and testing of composite panels, meticulously analyzing their tensile strength, flexural strength, and impact resistance^43,44^. This multifaceted research not only harnesses an abundant national resource and minimizes environmental waste but also opens new economic avenues, significantly contributing to environmental preservation^45^, and exploring the material’s vast potential for various composite applications^46,47^.

The intrinsic characteristics of areca fibers, including their crystalline architecture, physical dimensions, and bulk density, are fundamentally influenced by the plant’s stage of growth, the specific anatomical source (such as the outer husk or the inner leaf sheath), and the chosen fiber extraction methodology—with chemical processes often leading to enhanced structural order.

For these fibers to be effectively integrated into composite materials, a substantial alteration of their surface properties is imperative. Common modification techniques like alkalization, acetylation, and silanization are employed to remove non-cellulosic components. This process subsequently improves surface roughness, minimizes moisture absorption, and crucially maximizes the interfacial adhesion between the fiber and the matrix material. While combining different treatment strategies (hybrid approaches) can offer synergistic advantages, they frequently introduce increased practical complexity and higher costs. Similarly, cleaner physical methods, such as plasma treatment, are viable but generally expensive. Conversely, chemical modification routes, despite their efficacy, demand robust and often costly waste management systems due to the use of hazardous reagents.

These surface treatments primarily aim to reduce the content of hemicellulose, waxes, and lignin within the areca fibers, thereby improving their wettability and enhancing their bond with the composite matrix. Alkaline treatments are particularly effective at stripping away impurities and creating a rougher surface, which aids in mechanical interlocking. Acetylation works by reducing the fiber’s water absorption, making it more compatible with non-polar matrix materials. Silane treatments, on the other hand, establish a chemical bridge that strengthens the bond. Ultimately, these modifications expose more reactive cellulosic components, leading to superior interfacial bonding.

A significant challenge lies in achieving reproducible mechanical data for areca fibers. This difficulty arises from natural variations in the plant’s age, its specific component source, and diverse growing conditions. The problem is compounded by inconsistent reporting of critical treatment parameters, such as alkali concentration and exposure duration, across different studies. Further hindering direct comparisons is the sporadic documentation of fiber geometry, matrix properties, and fabrication techniques. Consequently, rigorous statistical analysis and comprehensive, meticulous reporting of all experimental conditions are essential to ensure data reliability and comparability.

### Objectives of the present study

The Indian state of Karnataka provides global researchers an optimal environment for pioneering work with areca fibers, offering a distinct convergence of cost-effectiveness, accessibility, and a well established scientific infrastructure. However, comprehensive comparative studies correlating the intrinsic physico-chemical properties of areca fibers—particularly those sourced from Karnataka—to their performance in composite systems are notably scarce. This study, therefore, aims to bridge this gap by meticulously integrating detailed fiber characterization with practical application assessment. Specifically, it will assess the physical, mechanical, and chemical properties of areca fibers, compare them with other natural fibers, and ultimately establish their viability as a reinforcing material for bio-based polymer composites in lightweight automotive components.

## Materials and methods

This section provides an overview of the processes involved in fiber separation, surface modification of fibers, chemical analysis, mechanical property assessment, and SEM analysis.

### Fiber separation

**Fig. 2:**
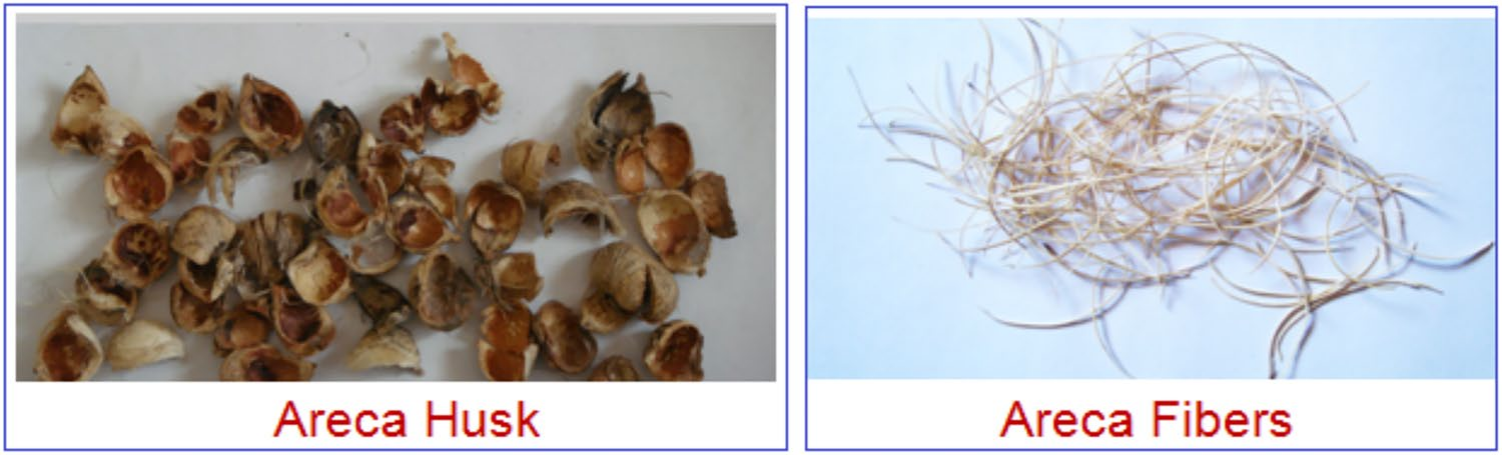
Areca husk and the separated areca fibers.

Extracting areca fibers, which are not naturally fine like some other natural fibers, necessitates mechanical processing from the husk. The process initiated with collecting areca nuts from local farms in Davangere, Karnataka, India. First, husks were removed from the nuts, dried, and then rehydrated via four days of water immersion. This soaking was critical for loosening the fibers and facilitating easier extraction. Next, the loosened fibers were rinsed with clean water and dried. The preliminary separation of areca fibers is achieved through a hammer mill, followed by further refinement using cyclone separators. This mechanical extraction method, however, carries a risk of fiber damage. Such degradation can manifest as diminished fiber length, alterations to key characteristics like tensile strength and aspect ratio, and an increased propensity for water absorption. To mitigate these detrimental effects and safeguard fiber quality, it is crucial to optimize the operational parameters of the hammer mill, including its gap and speed, and to utilize an appropriate hammer configuration. Furthermore, adjusting cyclone settings to facilitate a gentler separation process is vital. Research also indicates that pre-treatment strategies, such as alkali processing, can significantly improve fiber attributes and lessen processing-induced harm^48–50^. Figure 2 shows the areca husk and the separated fibers. Post-separation, the fibers were cleaned, washed, and finally sun-dried for 2–3 days to eliminate all moisture. For physical property analysis, 100 randomly selected dried areca fibers had their lengths measured.

### Fiber surface modification

Alkaline modification, primarily utilizing sodium hydroxide (NaOH) solutions, effectively purges non-cellulosic constituents like lignin, hemicellulose, and inorganic ash from fibers. This purification process inherently boosts the relative cellulose content within the fibers while simultaneously enhancing their surface topography. These chemically induced alterations are crucial for fostering superior interfacial adhesion and integration between the modified fibers and their encapsulating polymer matrices. Consequently, composites incorporating these treated fibers typically display elevated interfacial shear strength, enhanced resistance to impact, and optimized performance under both tensile and flexural loads. An additional observed benefit is a decrease in the material’s propensity for moisture uptake^51–53^. It is vital to note, however, that excessive NaOH concentrations or prolonged treatment durations risk inducing internal stress, micro-cracks, and overall fiber degradation, thereby causing general deterioration of the fiber structure^54–59^. Therefore, ongoing research is imperative to precisely define the optimal processing parameters—including the exact NaOH concentration, duration of exposure, and treatment temperature—in order to fully leverage the positive effects while concurrently mitigating any potential adverse consequences. Following the chemical modification, a comprehensive rinsing protocol is indispensable to ensure the complete removal of any residual processing agents. However, our current study, uniquely involved a 24-h immersion of areca fibers in a 15% sodium hydroxide (NaOH) solution. This alkaline treatment enhanced the fibers’ surface reactivity, promoting a uniform profile and improving compatibility for integration into polymer matrices.

### Chemical analysis

The chemical analysis of natural areca fiber was performed at the chemical laboratory of Harihar Polyfibers Ltd, a leader in pulp manufacturing in India. The chemical composition of the fibers was determined using the following methods: holocellulose was measured by the Sodium Chloride method, lignin according to TAPPI T 13 m-54, ash content followed TAPPI T 211 om-03, and moisture content assessed by TAPPI T 258 om-85.

### Mechanical property testing

The mechanical integrity of areca fibers was quantitatively evaluated through an advanced Hounsfield tensile testing system, detailed as follows.

This apparatus originates from Hounsfield, a leading British innovator in analytical instrumentation. Their sophisticated digital tension measurement systems are engineered for meticulous material characterization, leveraging an integrated, bespoke, Windows-operated software suite. Key functionalities encompass automated data processing, dynamic real-time graphical representation of test curves, and exhaustive data archival. This combination guarantees remarkable precision and strict conformity to global quality benchmarks across a wide spectrum of materials.

The loading platform’s movements are precisely orchestrated by a conventional personal computer, interfacing through a dedicated microprocessor. This configuration enables a flexible force application spectrum, ranging from 0.1 N to 50 N, along with user-configurable deformation rates spanning 5 to 200 mm/min. The intuitive software environment facilitates internal storage of all experimental outcomes, including visual representations, and allows for external export and post-testing modification of the data.

Within the scope of the current investigation, individual areca fibers were meticulously secured within a custom-fabricated paperboard jig. This jig featured a central aperture, specifically designed to obviate any twisting or fracturing of the fiber while it was firmly gripped by the machine’s jaws. Each fiber was then subjected to tensile loading at a uniform extension rate of 5 mm/min, with the effective gauge length set at 20 mm. The parameters assessed during this evaluation encompassed ultimate tensile strength, Young’s modulus (elastic modulus), and the elongation observed at the point of material failure.

### SEM analysis

Scanning Electron Microscope (SEM) images are essential for characterizing fiber structure and properties, especially when assessing suitability for natural fiber-reinforced polymer composites. These images provide insights into crucial features such as fiber diameter, surface roughness, and the presence of defects or voids^16,60^. Beyond individual fiber characteristics, SEM imaging also aids in evaluating fiber-matrix adhesion, fiber distribution, and the overall composite microstructure, all of which directly influence the composite’s mechanical and physical properties. In this study, images of both the surfaces and cross-sections of untreated areca fibers were captured using a high-resolution Cambridge Instruments electron microscope. This focus on untreated fibers was crucial for establishing a baseline understanding of their inherent characteristics before any modifications or treatments.

## Results and discussion

This section examines the physical and chemical properties of areca fibers, as well as the mechanical characteristics and dimensional changes associated with alkaline treatment of these fibers.

### Physical and chemical properties of areca fibers

This section deals with the comparison of physio-chemical properties and constituent elements of areca fibers with other natural fibers to establish areca fiber’s viability as a sustainable, eco-friendly, and lightweight alternative to synthetic fibers, providing a basis for its use in composites. The quantification of density in areca fibers was achieved by ascertaining their respective mass and spatial volume, a relationship fundamentally defined by: Density = Mass /Volume. For each discrete bundle of fibers, mass readings were obtained with an analytical balance, providing measurements precise to 0.01 g. The definitive mass figure attributed to any given bundle represented the arithmetic mean derived from numerous individual test specimens. Volume, conversely, was simply computed as the product of the sample’s linear dimension (length) and its transverse surface area.

The lengths of the selected areca fibers were were measured using a scale. The maximum length recorded was approximately 45 mm, while the minimum length was 32 mm. From the random measurements of areca fibers, the average length was found to be 35 mm. The diameter of the fibers was measured at three different points along their length using a micrometer, revealing a range of 200 to 550 $\mu m$. Consequently, the average diameter of the areca fibers is approximately 300 $\mu m$. The density of the areca fibers was calculated, and it was determined that the density values range from 1.230 to 1.250 g/cm^3^, with an average density of about 1.2435 g/cm^3^. The thickness of the cell walls, as observed through fiber morphology, ranges from 4 to 4.3 µm. A comparison of the physical properties of areca fibers with those of some other natural fibers can be found in Table 1. The chemical properties of areca natural fibers were analyzed and compared with the composition of selected samples, as presented in Table 2. Areca fibers exhibit high cellulose content. The total cellulose (Holocellulose) level in the fibers was determined to be 68%, which is greater than that of coir and banana fibers, but slightly lower than that of sisal. Additionally, the areca fiber exhibits 4.62% ash content. These characteristics enhance the fiber’s performance and its capability to reinforce polymers. The moisture content in the fiber was measured at 11.2%.

**Table 1:** Physical properties of natural fibers.

Physical properties	Sisal^61^	Banana^61^	Coir^61^	Areca
Diameter (µm)	100–300	50–250	100–450	200–550
Cell length to width ratio	100	150	35	95.36
Density (g cm^-3^)	1.45	1.35	1.15	1.243
Cell Wall thickness (µm)	12.5	1.25	8.0	4–4.3

**Table 2:** Chemical composition of selected samples.

Chemical composition	Sisal^62^	Banana^62^	Coir^63^	Areca
Cellulose (%)	66–72	63–64	32 – 43	53.2
Hemicellulose (%)	12	19	0.15 – 0.25	30.24
Lignin (%)	10–14	5	40 – 45	13
Ash (%)	–	–	–	4.62

### Alkaline treatment of areca fibers

Areca fibers contain various impurities, including wax, fatty substances, and globular protrusions known as “tyloses”. The purpose of the treatment is primarily to eliminate these impurities from the natural areca fibers, enhancing their versatility. While the washing process was effective in removing most impurities, it was unable to eliminate the protrusions. In contrast, alkali treatment successfully eliminated fatty deposits and tyloses, resulting in a roughened fiber surface with pits. This texture improves mechanical anchorage and decreases the likelihood of pullout and gaps within the fiber. Additionally, alkali treatment exposed a greater amount of cellulose on the fiber surface, thereby increasing the number of available reaction sites. Consequently, this treatment produces a cleaner fiber surface.

$$Fiber-OH+NaOH\Rightarrow Fiber-{O}^{-}N{a}^{+}+{H}_{2}O$$

**Fig. 3:**
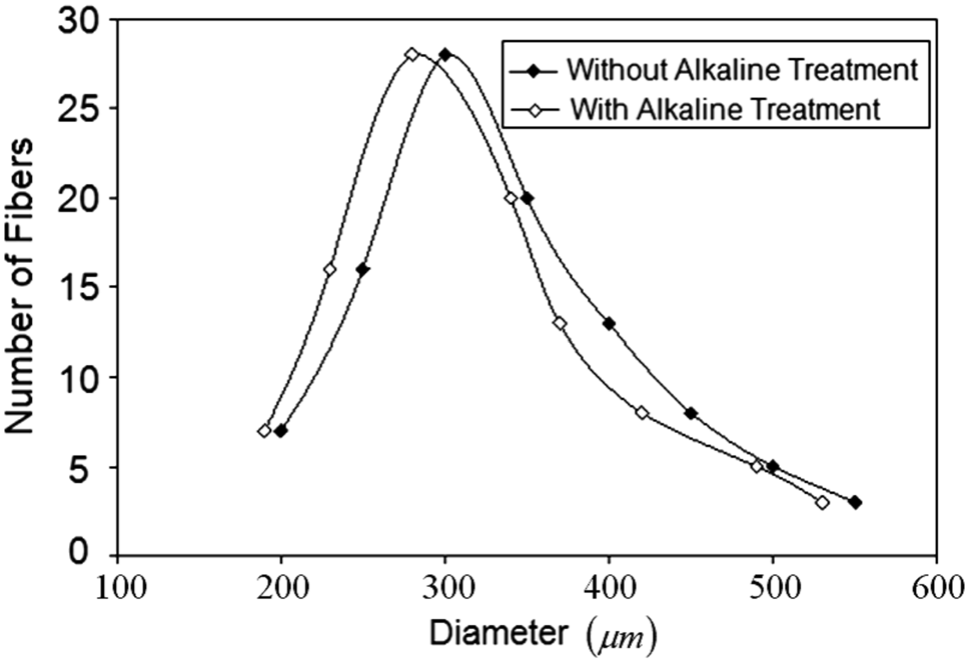
Variation of fiber diameter of treated and untreated areca fiber.

The changes in dimensions of the areca fibers following treatment are illustrated in the distribution curve presented in Fig. 3. The diameters of approximately 100 fibers were measured both before and after treatment, leading to the creation of the distribution curve. The chemical treatment caused a significant decrease in fiber diameter, with a reduction of 6–7%. This process likely removes the surface wax-type pith, resulting in a considerable reduction in diameter. Consequently, this leads to an increase in the aspect ratio, which enhances the mechanical properties of the composites^64^. Various chemical reactions during the treatment were reported by studies^65,66^.

### Mechanical properties

**Fig. 4:**
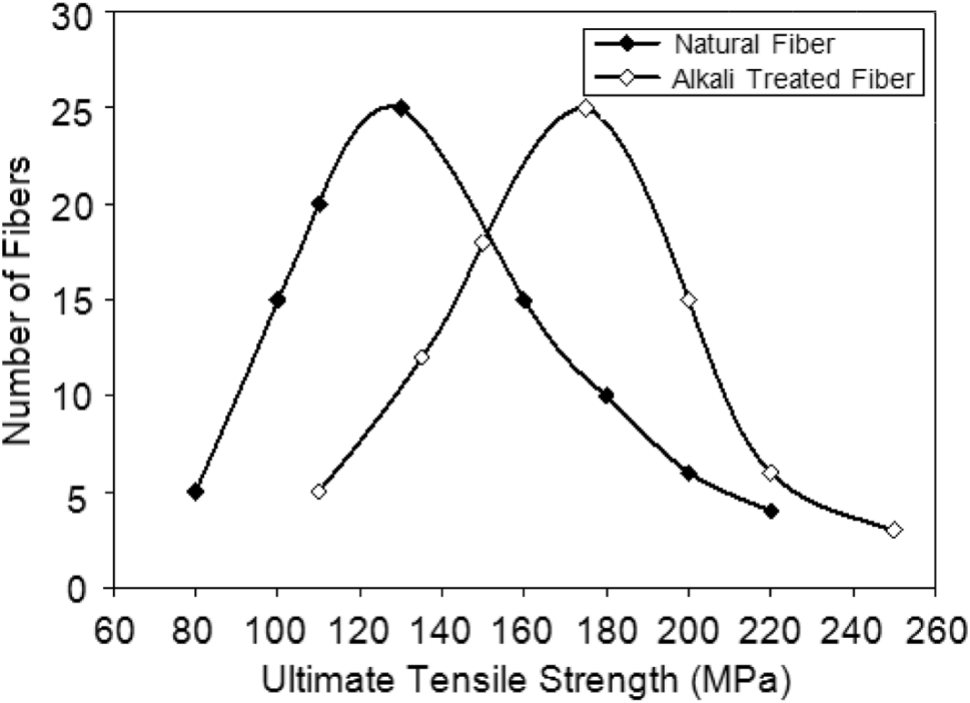
Variation of ultimate tensile strength (${\sigma }_{ult}$) of natural and alkali treated fibers.

The mechanical properties, particularly the ultimate tensile strength $\left({\sigma }_{ult}\right)$ of the samples, were evaluated using a computerized Tinius Olsen universal testing machine, following ASTM E4 standards. Tensile tests were carried out at room temperature on both treated and untreated areca fiber specimens, with a total of 100 samples assessed. Figure 4 illustrates the distribution curve for determining the mean ${\sigma }_{ult}$ of the areca fibers, revealing that the treatment positively affects the fibers’ strength. The maximum ${\sigma }_{ult}$ for the alkali-treated areca fibers is 175 ± 2.14 MPa, while the Young’s Modulus $\left(E\right)$ is 4.3 GPa, with elongation percentages (*%el*) ranging from 8 to 20%. In contrast, the ${\sigma }_{ult}$ of the untreated natural fibers is 130 ± 17.5 MPa, with *%el* ranging from 5 to 15%. The Young’s modulus $\left(E\right)$ for the average natural fiber is 3.95 GPa.

**Fig. 5:**
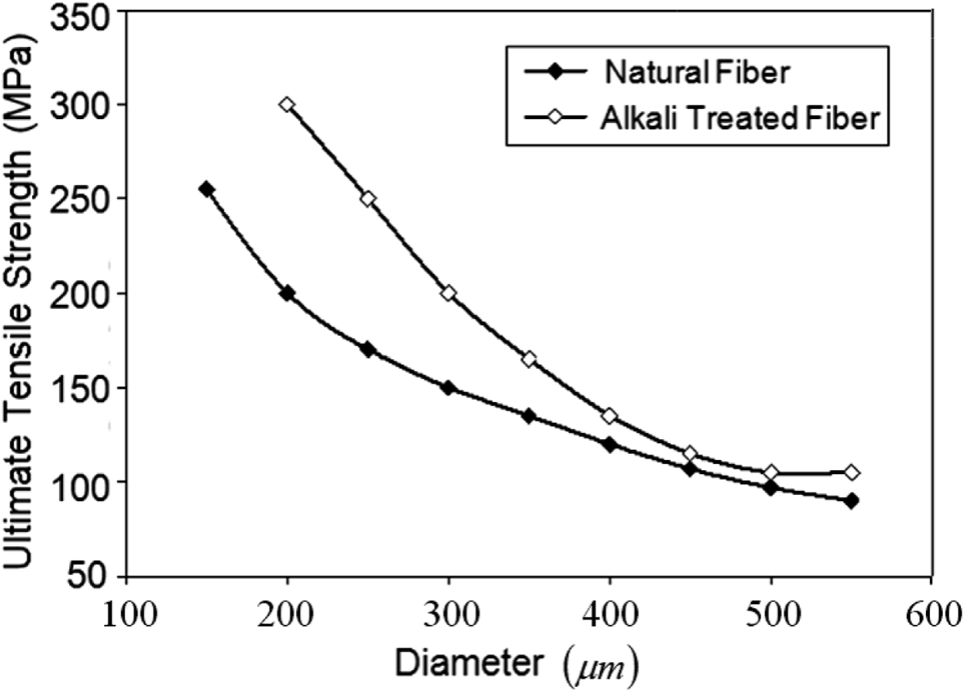
Comparison of the ultimate tensile strength (${\sigma }_{ult}$) with diameter of natural and alkali treated areca fibers.

Figure 5 illustrates the ultimate tensile strength (${\sigma }_{ult}$) of alkali-treated fibers compared to natural areca fibers across various diameters. It is evident that the ${\sigma }_{ult}$ is significantly influenced by fiber diameter, and a wide range of diameters exists within the same fiber bundle, highlighting a common limitation of natural fibers. ${\sigma }_{ult}$ decreases as the diameter of the fibers increases. For fibers with a consistent diameter of 300 $\mu m$, ${\sigma }_{ult}$ of natural areca fiber is 140 ± 17.5 MPa, whereas alkali-treated areca fibers exhibit strength of 175 ± 2.14 MPa. This indicates that alkali treatment enhances ${\sigma }_{ult}$ for fibers of the same diameter. Additionally, fibers from different plant sources may vary in nature and texture. The characteristics of the fibers, including diameter and density, influence their properties, resulting in substantial variations in the observed data. Consequently, an average value of these properties is provided.

### Microfibrillar angle and fiber strength

The strength of fibers is influenced by their fibrillar structure, the microfibrillar angle (*θ*), and the cellulose content (W). The *%el* expressed in terms of *θ* is^67^

1 $$\%el=-2.78+7.28\times 1{0}^{-2}\times \theta +7.7\times 1{0}^{-3}\times {\theta }^{2}$$

According to Eq. (1), the microfibrillar angle (*θ*) in terms of *%el* is

2 $$\theta =11.396\times \sqrt{2.9521+\%el}-4.7273$$

Furthermore, ${\sigma }_{ult}$ related to *θ* and W is^67^

3 $${\sigma }_{ult}=-334.005-2.83\times \theta +12.22\times W$$

To assess the sufficiency of relationships (1) to (3), Table 3 displays a comparison between measured and estimated Cellulose content (W) of various fibers. The microfibrillar angle (*θ*) is calculated using Eq. (2) based on the measured percentage elongation. The W is derived from Eq. (3) utilizing the determined *θ* from *%el* and ${\sigma }_{ult}$. Variations in W values between the measured and estimated results could be attributed to the natural properties of the fiber’s cellular structure and the empirical relationships outlined in (1) to (3).

**Table 3:** Comparison of measured and estimated Cellulose content (W) from the mechanical properties of different fibers.

Fiber	Ultimate tensile strength, ${\sigma }_{ult}$ (MPa)	Percentage elongation, *%el*	Microfibrillar angle, $\theta$ (degree) Eq. (2)	Cellulose content, W (%)	
				Measured	Estimate Eq. (3)
Sisal^55^	580	4.3	25.96	66–72	80.81
Banana^55^	540	3.0	23.08	63–64	76.87
Coir^55^	140	25	55.52	32–43	55.52
AHF^19^	145.48–151.16	3.86–10.44	25.02–36.98	52.37–55.93	45.03–48.27
AHF-1^19^	147–322	10.23–13.15	36.65–41.04	57.35–58.21	47.85–63.19
AHF-2^19^	123.93–166.03	22.56–23.76	52.83–54.17	53.20	49.71–53.46

**Fig. 6:**
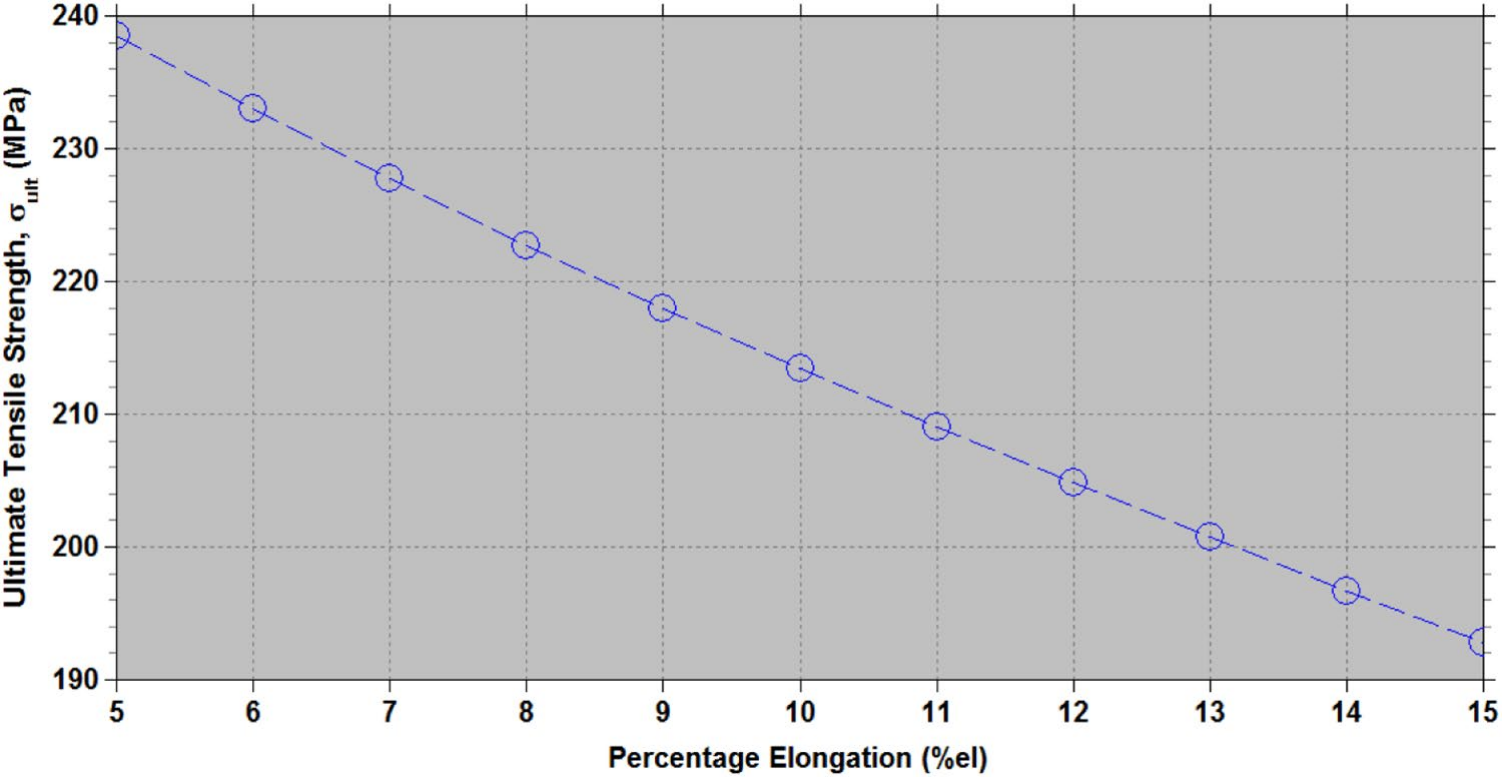
Variation of the ultimate tensile strength (${\sigma }_{ult}$) untreated areca husk fiber with percentage elongation (*%el*) using Eqs. (2) and (3) and specifying the Cellulose content, W = 53.2%.

In the current study, the *%el* of untreated areca husk fiber (AHF) ranges from 5 to 15%. $\theta$ as derived from Eq. (2), changes from 27.41° to 43.56 ^o^. Figure 6 illustrates the ultimate tensile strength (${\sigma }_{ult}$) of untreated areca husk fiber determined from Eqs. (2) and (3) for a Cellulose content (W) of 53.2%, and varying the percentage elongation (*%el*).

This analysis reveals that as the *%el* increases, the microfibrillar angle (*θ*) also increases, leading to a corresponding decrease in the tensile strength (${\sigma }_{ult}$). Hence, ${\sigma }_{ult}$ varies between 192.8 to 238.5 MPa, while the experimental data show values ranging from 112.5 to 147.5 MPa. This difference suggests that the estimated strength is greater than the experimental results, likely due to the intrinsic properties of the fiber’s cellular structure or the empirical relationships used in the calculations.

Rekha and Nagaraja Ganesh^68^ reported that the microfibrillar angle ($\theta$) of *Cocos nucifera* fibers, determined by X-ray diffraction, varied from 26.54° to 27.78^o^. Their tensile tests revealed ${\sigma }_{\mathrm{ult}}$ values ranging from 132.45 to 196.72 MPa and *%el* from 12.1 to 17.6%. Using the global strain equation: $\theta =\frac{180}{\pi }{\mathit{sec}}^{-1}\left\{1+\frac{\%el}{100}\right\}$, they calculated $\theta$ values ranging from 26.87° to 31.75 ^o^. They also reported $\theta$ values from the literature, which varied between 30° and 49°^57,58^. Furthermore, $\theta$ values for the measured *%el* (from 12.1 to 17.6%), when calculated using Eq. (2), ranged from 39.49° to 46.94°. Notably, the $\theta$ values obtained using the global strain equation were approximately 66% of those derived from Eq. (2). The discrepancies observed among $\theta$ values derived from X-ray diffraction, the global strain equation, and those reported in literature can be attributed to several factors. These include the characteristics of the dry, mature cellulosic fibers extracted from *Cocos nucifera* fruit, the non-uniformity of the fiber cross-section, and external pulling forces capable of reorganizing the amorphous and crystalline contents within the cell wall. Nevertheless, the X-ray diffraction technique, as detailed in^68–70^, offers a simple, cost-effective, and acceptably accurate method, notably without cumbersome processing.

### Morphological analysis of natural areca fibers

Alkali treatment profoundly alters the functional groups and chemical bonds of natural fibers. To evaluate the effect of sodium hydroxide (NaOH) treatment on Areca fibers, Fourier-transform infrared (FTIR) spectroscopy was performed in transmittance mode (500–4000 cm⁻^1^)^71–73^, comparing the spectra of both untreated and treated fibers.

**Fig. 7:**
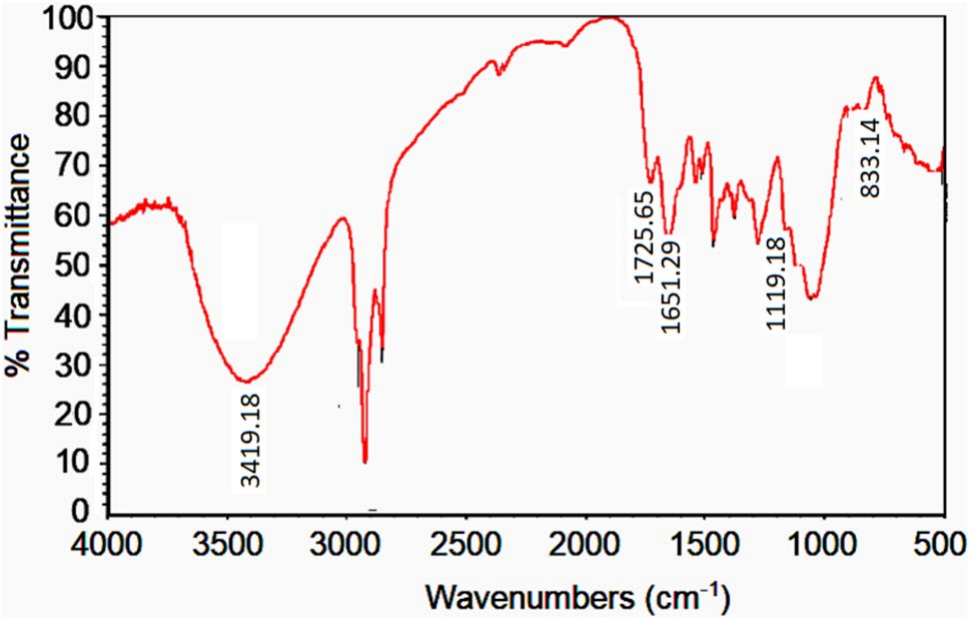
FTIR Spectrum of untreated areca fiber^71^.

**Fig. 8:**
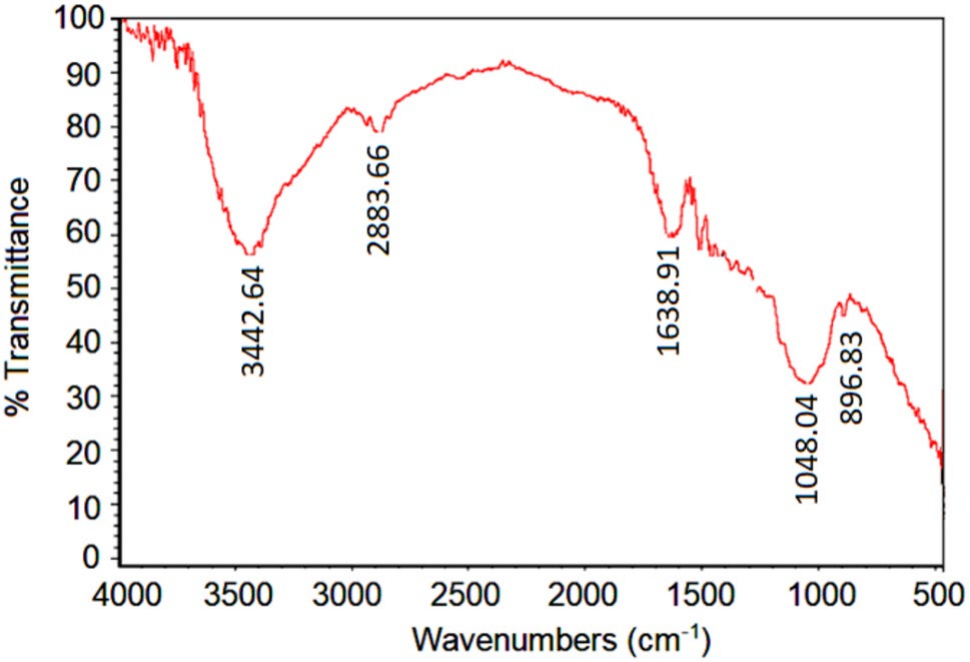
FTIR Spectrum of alkali treated areca fiber^71^.

Untreated Areca fibers exhibited characteristic FTIR peaks: O–H stretching at 3419.18 cm⁻^1^, C-O stretching between 1040 and 1060 cm⁻^1^, ester/ether crosslinks at 1727.65 cm⁻^1^, hemicellulose in the 1100–1600 cm⁻^1^ range, and cellulose alcohol at 1377.86 cm⁻^1^ (see Fig. 7). Following alkali treatment, the complete disappearance of the 1727.65 cm⁻^1^ peak indicated the hydrolysis of ester and ether bonds (see Fig. 8). Furthermore, a reduced intensity of the 1100–1600 cm⁻^1^ peaks suggested the partial removal of hemicelluloses from the fiber surface.

**Fig. 9:**
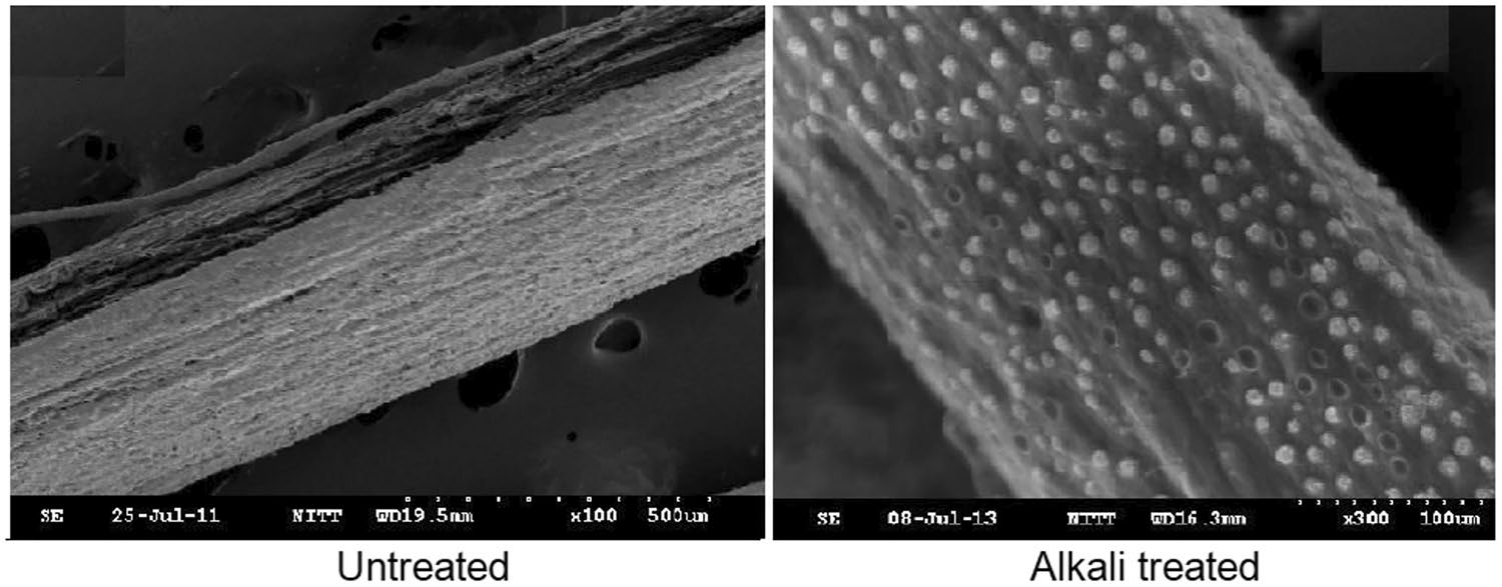
SEM images of areca fiber^71^.

Scanning Electron Microscope (SEM) analysis revealed substantial topographical changes in alkali-treated fibers compared to their untreated counterparts (see Fig. 9). This transformation is attributed to the removal of low molecular weight compounds, leading to a significant increase in surface roughness. Consequently, this enhanced surface roughness contributes to decreased moisture absorption and provides a larger surface area for superior adhesion with the matrix^71^.

**Fig. 10:**
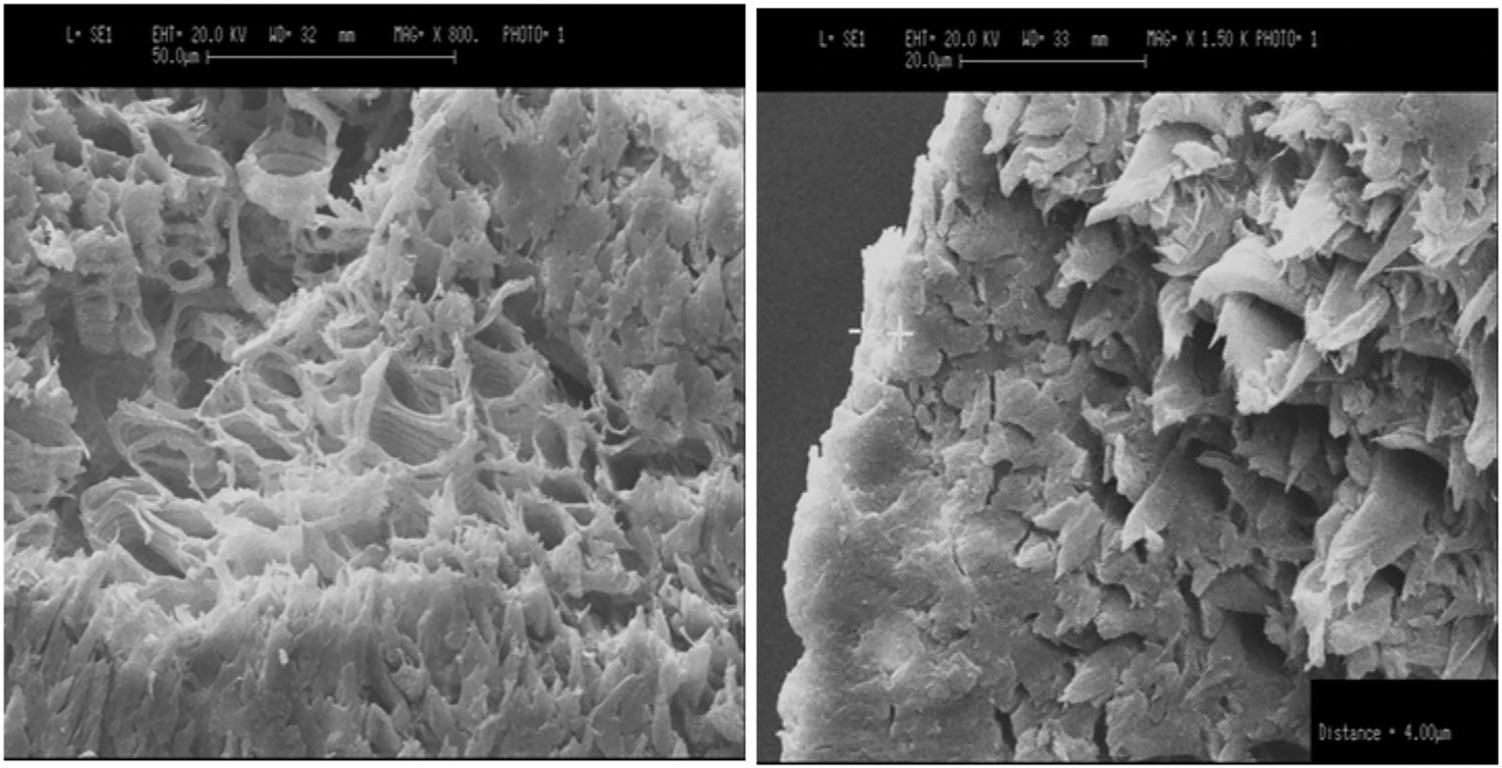
SEM image of fracture surface of natural areca fiber.

The SEM image in Fig. 10 specifically illustrates a rough and uneven fracture surface in the treated fibers. This texture is advantageous as it offers an expanded surface area for the matrix material to adhere to, thereby promoting a stronger fiber-matrix bond. Additionally, the image highlights a distinctive honeycomb-like internal structure within the areca fiber. This structure, characterized by small lumens (hollow spaces) and irregularly spaced nodes, divides the fiber into distinct cells. Such internal morphology can significantly influence the fiber’s mechanical properties and its interaction within a composite matrix.

### Influence of diameter and length of areca fiber on tensile strength

Binoj et al.^32^ have investigated how the diameter (${D}_{af}$) and length (${L}_{af}$) of Areca fruit husk fiber (AFHF) influence its strength characteristics. For their experimental design, they utilized Taguchi’s L_25_ orthogonal array (OA), exploring five diameters that range from 300 to 500 μm in 50 μm increments, as well as gauge length from 10 to 50 mm, increasing in 10 mm steps. In addition, they implemented a full factorial design for the experiments. The highest tensile strength recorded for AFHF was 223.3 MPa at a diameter of ${D}_{af}$=400 μm and a length of ${L}_{af}$=40 mm. Taguchi’s design philosophy allows for fewer experiments while still producing sufficient data for a comprehensive factorial analysis. This study aims at assess the capabilities of the modified Taguchi method^74–78^ by utilizing Taguchi’s L_9_ OA for the two parameters (${D}_{af}$ and ${L}_{af}$) each with three levels. Table 4 presents the levels of these physical parameters (${D}_{af}$ and ${L}_{af}$). The tensile properties of AFHF corresponding to the levels of ${D}_{af}$ and ${L}_{af}$ as outlined in Table 5, follow the configuration laid out by Taguchi’s L_9_ OA. Additionally, the ANOVA results in Table 6 illustrate the contribution of ${D}_{af}$ and ${L}_{af}$ to the overall mean values of the strength properties.

**Table 4:** Assigned levels of the physical parameters (${D}_{af}$ and ${L}_{af}$) of AFHF.

Physical parameters of AFHF	Designation	Level-1	Level-2	Level-3
Diameter ($\mu \hspace{0.33em}m$)	${D}_{af}$	300	400	500
Length (mm)	${L}_{af}$	10	30	50

**Table 5:** Strength properties of AFHF as per the Taguchi’s L_9_ OA.

Test Run	Levels		Strength properties					
	${D}_{af}$	${L}_{af}$	${\sigma }_{ult}\hspace{0.33em}(MPa)$ Test^32^	${\sigma }_{ult}\hspace{0.33em}(MPa)$ Eq. (3)	$E\hspace{0.33em}(GPa)$ Test^32^	$E\hspace{0.33em}(GPa)$ Eq. (4)	*%el* Test^32^	*%el* Eq. (5)
1	1	1	102.1	92.80	9.9	8.51	10.3	10.122
2	1	2	121.3	127.27	14.4	14.74	8.42	8.549
3	1	3	128.6	131.93	17.5	18.54	7.35	7.399
4	2	1	147.2	165.17	12.1	13.08	12.2	12.012
5	2	2	209.1	199.63	20.1	19.31	10.4	10.439
6	2	3	212.8	204.30	23.3	23.11	9.14	9.289
7	3	1	107.1	98.43	15	15.41	7.16	7.526
8	3	2	129.4	132.90	21.2	21.64	6.12	5.952
9	3	3	132.4	137.57	26.3	25.44	5.0	4.802

**Table 6:** ANOVA for strength properties of AFHF.

Properties	Physical parameters	1st mean	2nd mean	3rd mean	Average	Sum of squares (SOS)	% contribution
${\sigma }_{ult}(MPa)$	${D}_{af}$	117.33	189.70	122.97	143.33	9722.01	73.7
	${L}_{af}$	118.80	153.27	157.93	143.33	2741.15	20.8
$E(GPa)$	${D}_{af}$	13.933	18.500	20.833	17.756	73.91	31.6
	${L}_{af}$	12.333	18.567	22.367	17.756	153.96	65.9
*%el*	${D}_{af}$	8.690	10.580	6.093	8.454	30.44	72.5
	${L}_{af}$	9.887	8.313	7.163	8.454	11.21	26.7

In order to achieve the highest ${\sigma }_{ult}$, the optimal physical parameters identified in ANOVA Table 6 are ${D}_{a{f}_{2}}{L}_{a{f}_{3}}$ with subscripts signifying the levels of parameters ${D}_{af}$ and ${L}_{af}$. To estimate the strength characteristics, including ${\sigma }_{ult}$, Young’s modulus (*E*), and *%el*, the additive law^74^ is applied. In this scenario, involving two physical parameters (${D}_{af}$ and ${L}_{af}$), the strength characteristics (${\sigma }_{ult}$, *E*, and *%el*) are calculated using the mean values from ANOVA Table 6 in accordance with the additive law.

4 $$\sigma _{{ult}} = \left( {\bar{\sigma }_{{ult}} } \right)_{{D_{{af_{i} }} }} + \left( {\bar{\sigma }_{{ult}} } \right)_{{L_{{af_{j} }} }} - \sigma _{{ult_{{mean}} }} fori,j = 1to3$$

5 $$E = \left( {\bar{E}} \right)_{{D_{{af_{i} }} }} + \left( {\bar{E}} \right)_{{L_{{af_{j} }} }} - E_{{mean}} fori,j = 1to3$$

6 $$\%el={\left(\%el\right)}_{{D}_{a{f}_{i}}}+{\left(\%el\right)}_{{L}_{a{f}_{j}}}-\%e{l}_{mean}fori,j=1to3$$

The minimum and maximum discrepancies between the test results and the estimates derived from Eqs. (4) to (6) provide adjustments to the tensile strength characteristics of AFHF. According to Table 5, the adjustments to derive lower and upper bound values for Eq. (4) are −17.96 MPa and 9.47 MPa, respectively. For Eq. (5), the corresponding adjustments are −1.044 GPa and 1.389 GPa. Meanwhile, the corrections for Eq. (6) are -0.366 and 0.198%.

For the optimal physical parameters identified (${D}_{a{f}_{2}}{L}_{a{f}_{3}}$), the range of estimated ${\sigma }_{ult}$ from Eq. (4) after applying the corrections falls between 186.33 MPa and 213.77 MPa. The estimated range for *E*, derived from Eq. (5), following the corrections, is between 22.07 and 24.50 GPa. Additionally, the estimated range for *%el*, based on Eq. (6) after incorporating the corrections, is from 8.923 to 9.487%. The test data for the strength properties corresponding to the optimal physical parameters are: ${\sigma }_{ult}$=212.829 MPa; *E* = 23.285 GPa; and *%el* = 9.14%, all of which fall within the estimated range.

Based on the mean values of strength properties in ANOVA Table 6 for the levels of physical parameters (${D}_{af}$ and ${L}_{af}$), empirical relationships are formulated as follows.

7 $${\sigma }_{ult}=199.63+2.8167{\xi }_{1}-69.55{\xi }_{1}^{2}+19.5667{\xi }_{2}-14.9{\xi }_{2}^{2}$$

8 $$E=19.311+3.45{\xi }_{1}-1.1167{\xi }_{1}^{2}+5.0167{\xi }_{2}-1.2167{\xi }_{2}^{2}$$

9 $$\%el=10.439-1.2983{\xi }_{1}-3.1883{\xi }_{1}^{2}-1.3617{\xi }_{2}+0.2117{\xi }_{2}^{2}$$

Here,$${\xi }_{1}=\frac{1}{100}\left({D}_{af}-400\right)$$ and $${\xi }_{2}=\frac{1}{20}\left({L}_{af}-30\right)$$.

**Fig. 11:**
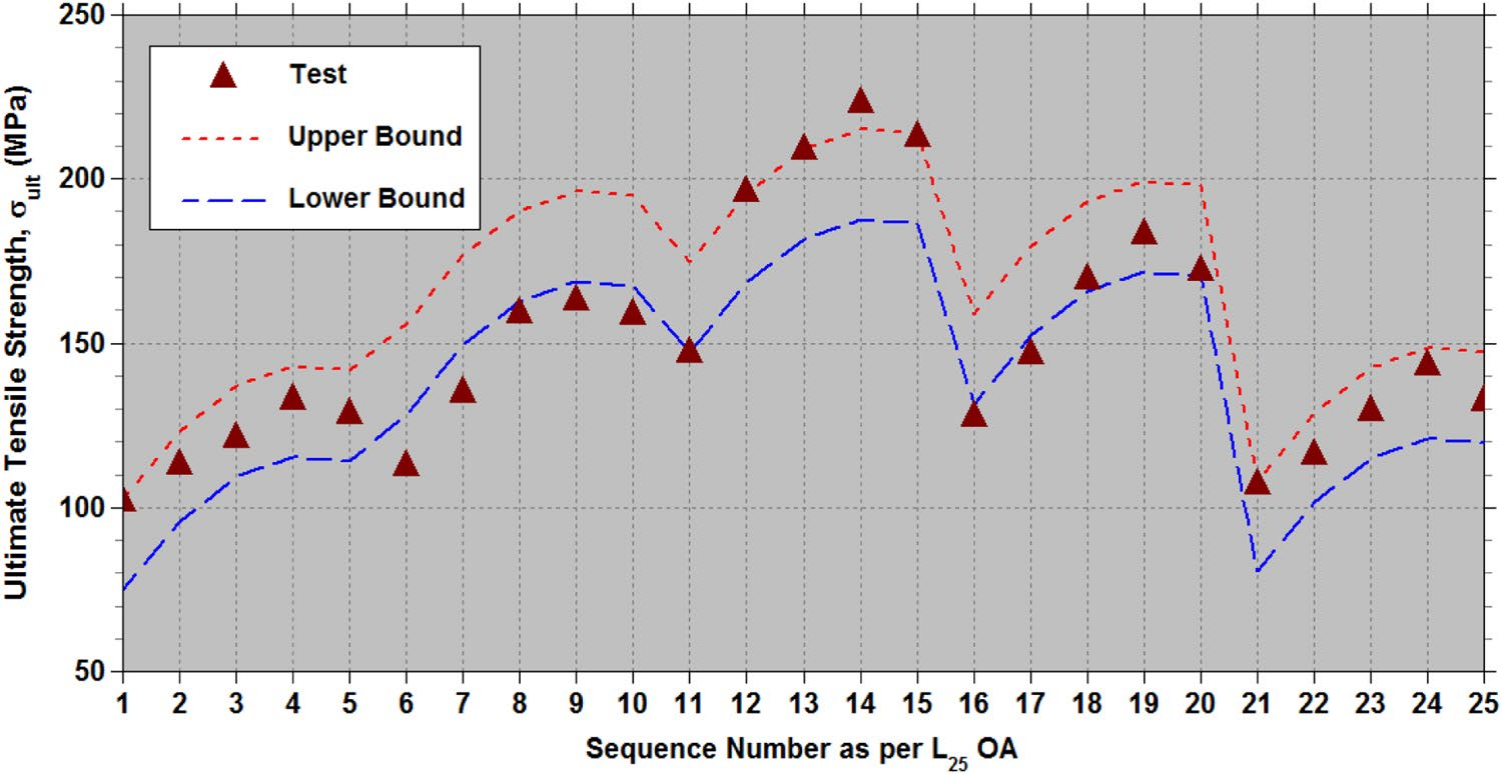
Lower and upper limit estimates of ${\sigma }_{ult}$ with test data of AFHF^32^.

**Fig. 12:**
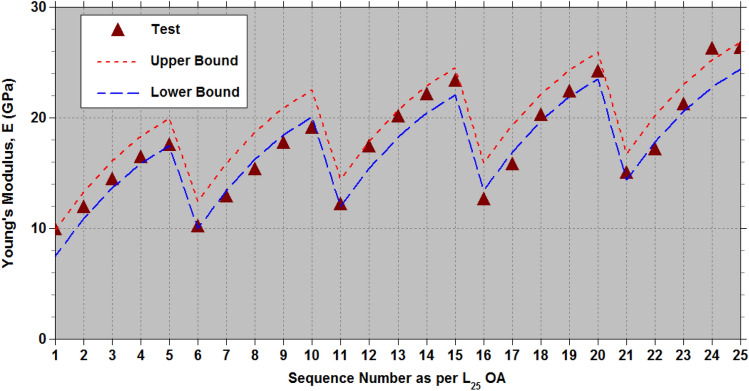
Lower and upper limit estimates of $E$ with test data of AFHF^32^.

**Fig. 13:**
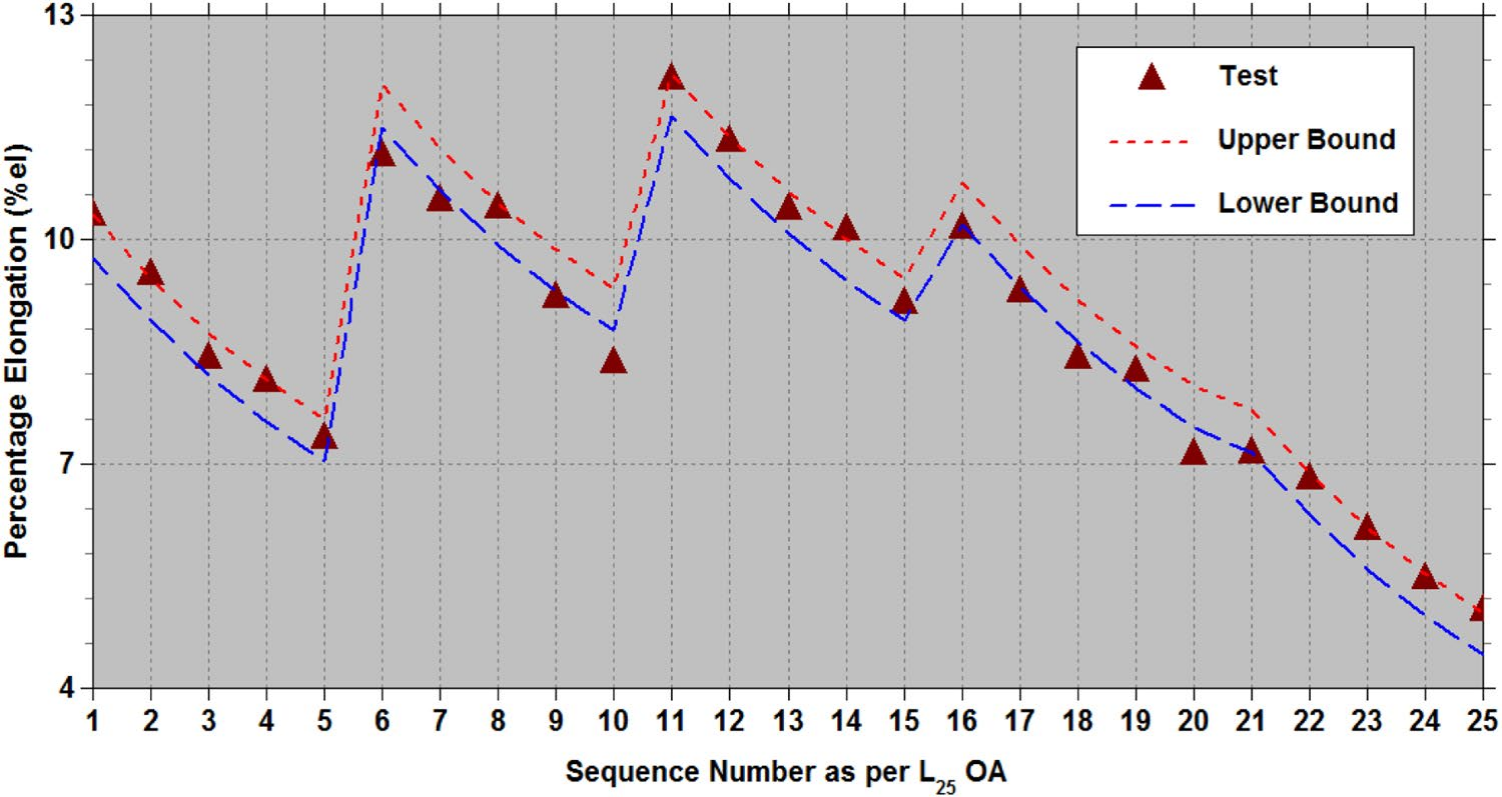
Lower and upper limit estimates of $\%el$ with test data of AFHF^32^.

The effectiveness of the established empirical relationships (7) to (9) is evaluated using the test data^32^, which includes physical parameter levels of ${L}_{af}\in$ (10 mm, 20 mm, 30 mm, 40 mm, and 50 mm) and ${D}_{af}\in$ (300 μm, 350 μm, 400 μm, 450 μm and 500 μm). This test data^32^ encompasses all 25 combinations of the two physical parameters (${D}_{af}$ and ${L}_{af}$) with five levels,$\left(\left(\left({D}_{a{f}_{i}},{L}_{a{f}_{j}}\right),j=1to5\right),i=1to5\right)$ generated according to the L_25_ OA at a constant cross-head speed of 5 mm/min under standard atmospheric conditions. Tables 7, 8, 9 (as well as Figs. 11, 12, 13 offer a fairly accurate comparison between the estimated strength properties derived from Eqs. (7) to (9) and the test data^32^ for L_25_ OA.

**Table 7:** Comparison of the estimated tensile strength (${\sigma }_{ult}$) with the test data from reference^32^ for L_25_ OA.

S. No	Physical parameters		Transformed parameters		Ultimate tensile strength,${\sigma }_{ult}\hspace{0.33em}(MPa)$			
	${D}_{af}$ ($\mu m$)	${L}_{af}$ (mm)	${\xi }_{1}$	${\xi }_{2}$	Test^32^	Eq. (7)	Lower bound	Upper bound
1	300	10	−1	−1	102.143	92.79	74.82	102.26
2	300	20	−1	−0.5	113.181	113.75	95.78	123.22
3	300	30	−1	0	121.321	127.26	109.29	136.73
4	300	40	−1	0.5	133.151	133.32	115.35	142.79
5	300	50	−1	1	128.623	131.93	113.96	141.40
6	350	10	−0.5	−1	112.673	146.36	128.39	155.83
7	350	20	−0.5	−0.5	135.134	167.32	149.35	176.79
8	350	30	−0.5	0	159.363	180.83	162.86	190.30
9	350	40	−0.5	0.5	163.122	186.89	168.92	196.36
10	350	50	−0.5	1	158.843	185.50	167.53	194.97
11	400	10	0	−1	147.215	165.16	147.19	174.63
12	400	20	0	−0.5	196.124	186.12	168.15	195.59
13	400	30	0	0	209.123	199.63	181.66	209.10
14	400	40	0	0.5	223.321	205.69	187.72	215.16
15	400	50	0	1	212.829	204.30	186.33	213.77
16	450	10	0.5	−1	127.871	149.18	131.21	158.65
17	450	20	0.5	−0.5	146.653	170.14	152.17	179.61
18	450	30	0.5	0	169.621	183.65	165.68	193.12
19	450	40	0.5	0.5	183.522	189.71	171.74	199.18
20	450	50	0.5	1	172.192	188.32	170.35	197.79
21	500	10	1	−1	107.145	98.43	80.46	107.90
22	500	20	1	−0.5	116.346	119.39	101.42	128.86
23	500	30	1	0	129.418	132.90	114.93	142.37
24	500	40	1	0.5	143.219	138.96	120.99	148.43
25	500	50	1	1	132.394	137.57	119.60	147.04

**Table 8:** Comparison of the estimated Young’s modulus (*E*) with the test data from reference^32^ for L_25_ OA.

S. No	Physical parameters		Transformed parameters		Young’s modulus,$E\hspace{0.33em}(GPa)$			
	${D}_{af}$ ($\mu m$)	${L}_{af}$ (mm)	${\xi }_{1}$	${\xi }_{2}$	Test^32^	Eq. (8)	Lower bound	Upper bound
1	300	10	−1	−1	9.898	8.51	7.47	9.90
2	300	20	−1	−0.5	11.876	11.93	10.89	13.32
3	300	30	−1	0	14.409	14.74	13.70	16.13
4	300	40	−1	0.5	16.418	16.95	15.90	18.34
5	300	50	−1	1	17.500	18.54	17.50	19.93
6	350	10	−0.5	−1	10.132	11.07	10.03	12.46
7	350	20	−0.5	−0.5	12.833	14.49	13.45	15.88
8	350	30	−0.5	0	15.294	17.31	16.26	18.70
9	350	40	−0.5	0.5	17.673	19.51	18.47	20.90
10	350	50	−0.5	1	19.023	21.11	20.06	22.50
11	400	10	0	−1	12.116	13.08	12.03	14.47
12	400	20	0	−0.5	17.341	16.50	15.45	17.89
13	400	30	0	0	20.089	19.31	18.27	20.70
14	400	40	0	0.5	22.046	21.52	20.47	22.90
15	400	50	0	1	23.285	23.11	22.07	24.50
16	450	10	0.5	−1	12.598	14.52	13.48	15.91
17	450	20	0.5	−0.5	15.752	17.94	16.90	19.33
18	450	30	0.5	0	20.169	20.76	19.71	22.15
19	450	40	0.5	0.5	22.299	22.96	21.92	24.35
20	450	50	0.5	1	24.117	24.56	23.51	25.95
21	500	10	1	−1	14.964	15.41	14.37	16.80
22	500	20	1	−0.5	17.085	18.83	17.79	20.22
23	500	30	1	0	21.147	21.64	20.60	23.03
24	500	40	1	0.5	26.183	23.85	22.80	25.24
25	500	50	1	1	26.269	25.44	24.40	26.83

**Table 9:** Comparison of the estimated percentage elongation (*%el*) with the test data from reference^32^ for L_25_ OA.

S. No	Physical parameters		Transformed parameters		Percentage elongation (*%el*)			
	${D}_{af}$ ($\mu m$)	${L}_{af}$ (mm)	${\xi }_{1}$	${\xi }_{2}$	Test^32^	Eq. (9)	Lower bound	Upper bound
1	300	10	−1	−1	10.32	10.122	9.757	10.320
2	300	20	−1	−0.5	9.53	9.283	8.917	9.481
3	300	30	−1	0	8.42	8.549	8.183	8.747
4	300	40	−1	0.5	8.11	7.921	7.555	8.119
5	300	50	−1	1	7.35	7.399	7.033	7.597
6	350	10	−0.5	−1	11.12	11.864	11.499	12.062
7	350	20	−0.5	−0.5	10.53	11.025	10.659	11.223
8	350	30	−0.5	0	10.42	10.291	9.925	10.489
9	350	40	−0.5	0.5	9.23	9.663	9.298	9.861
10	350	50	−0.5	1	8.35	9.141	8.775	9.339
11	400	10	0	−1	12.15	12.012	11.647	12.210
12	400	20	0	−0.5	11.31	11.173	10.807	11.371
13	400	30	0	0	10.41	10.439	10.073	10.637
14	400	40	0	0.5	10.13	9.811	9.445	10.009
15	400	50	0	1	9.14	9.289	8.923	9.487
16	450	10	0.5	−1	10.15	10.566	10.201	10.764
17	450	20	0.5	−0.5	9.31	9.727	9.361	9.924
18	450	30	0.5	0	8.41	8.993	8.627	9.191
19	450	40	0.5	0.5	8.23	8.365	7.999	8.563
20	450	50	0.5	1	7.14	7.843	7.477	8.041
21	500	10	1	−1	7.16	7.526	7.160	7.724
22	500	20	1	−0.5	6.81	6.686	6.321	6.884
23	500	30	1	0	6.12	5.952	5.587	6.150
24	500	40	1	0.5	5.47	5.324	4.959	5.522
25	500	50	1	1	5.04	4.802	4.437	5.000

**Fig. 14:**
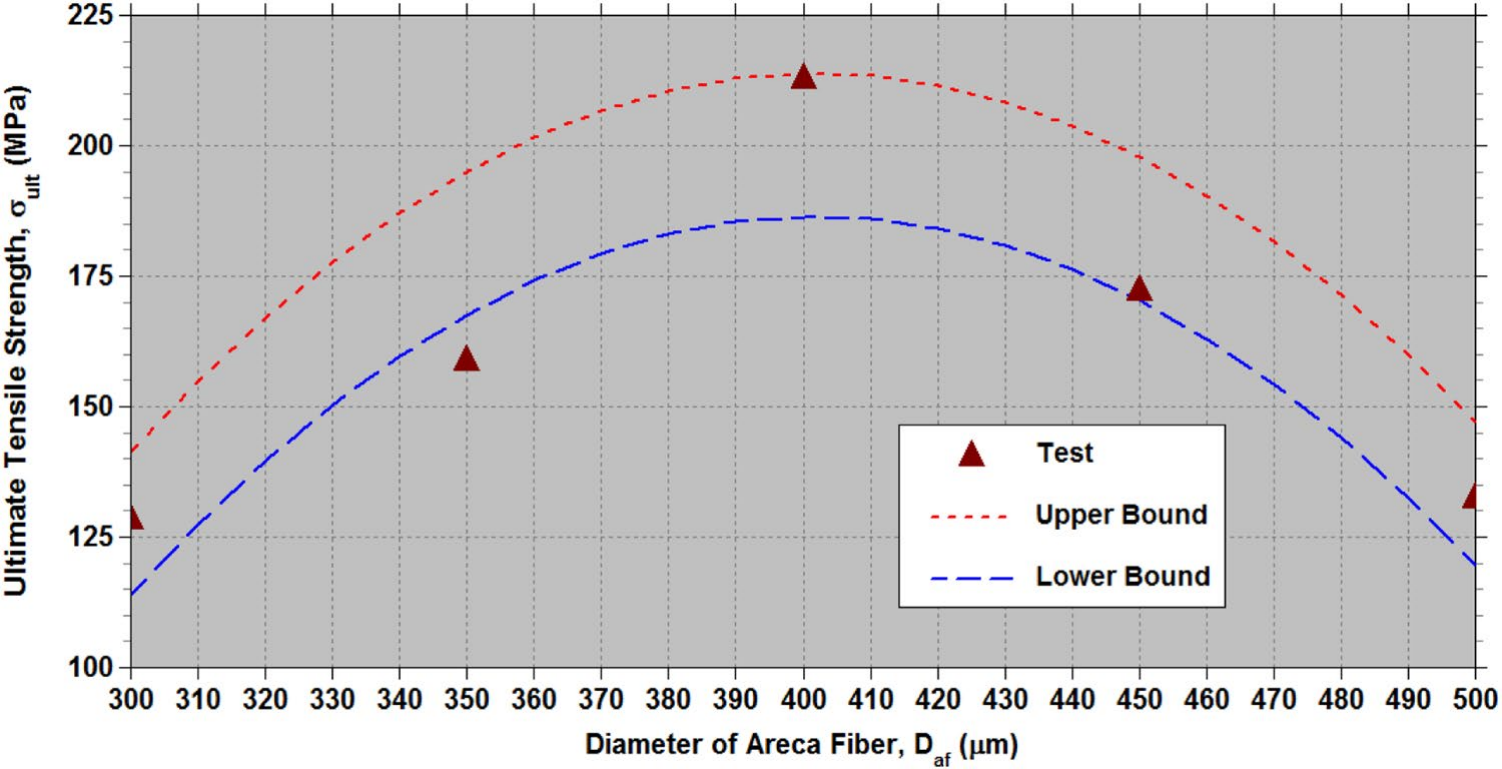
Variation of the ultimate tensile strength (${\sigma }_{ult}$) of AFHF with areca fiber diameter (${D}_{af}$) for the length of the areca fiber, ${L}_{af}$=50 mm.

**Fig. 15:**
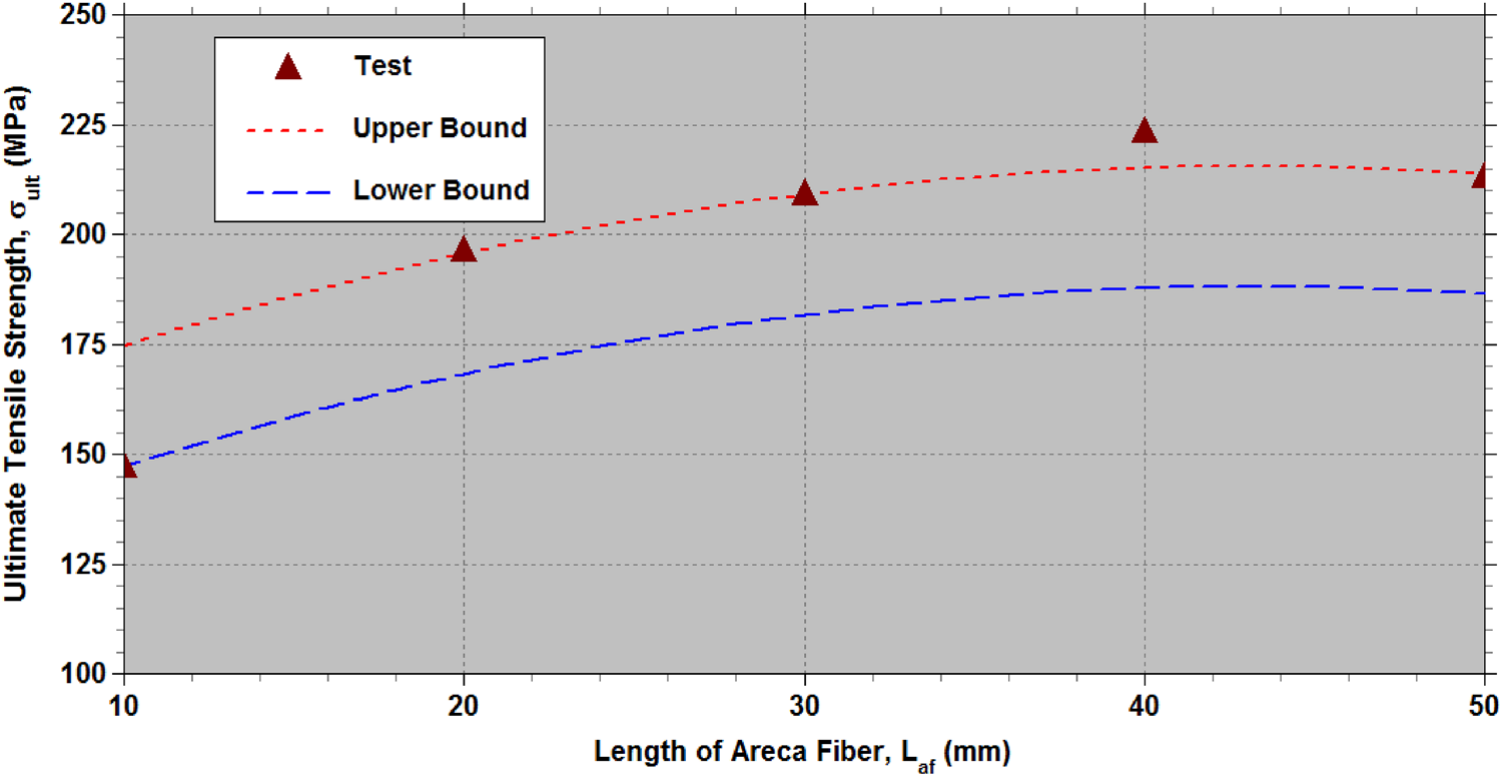
Variation of the ultimate tensile strength (${\sigma }_{ult}$) of AFHF with length of the areca fiber (${L}_{af}$) for the diameter of the areca fiber, ${D}_{af}$= 400 $\mu m$.

According to the ANOVA results presented in Table 6, the diameter (${D}_{af}$) is the most crucial physical parameter affecting ${\sigma }_{ult}$ of AFHF, when compared to fiber length (${L}_{af}$). To attain the maximum ${\sigma }_{ult}$, the optimal physical parameters are set at ${D}_{af}$= 400 $\mu m$ and ${L}_{af}$=50 mm. Figure 14 illustrates the relationship between ${\sigma }_{ult}$ and ${D}_{af}$ for ${L}_{af}$=50 mm. Meanwhile, Fig. 15 displays the relationship between *σ*_*ul*_ ${L}_{af}$ for ${D}_{af}$= 400 $\mu m$.

The optimal physical parameters for the maximum ${\sigma }_{ult}$ determined from the mean values of ANOVA Table 6 are at ${D}_{af}$= 400 $\mu m$ and ${L}_{af}$=50 mm, while the maximum ${\sigma }_{ult}$ from the tests^32^ in Table 6 correspond to ${D}_{af}$= 400 $\mu m$ and ${L}_{af}$=40 mm. A minor difference in values of ${\sigma }_{ult}$ is observed, as shown in Fig. 12, due to the low significance of ${L}_{af}$. In this study, the average length (${L}_{af}$) of the areca husk fibers was determined to be 35 mm, with a diameter (${D}_{af}$) of 300 $\mu m$. By utilizing these physical parameters in the empirical Eq. (7) and incorporating the necessary corrections, the calculated values of ${\sigma }_{ult}$ range from 113.25 to 140.69 MPa. In contrast, the experimental data revealed values between 112.5 and 147.5 MPa.

## Conclusions

This study characterizes areca fibers sourced from Karnataka, India, evaluating their potential as reinforcement for biopolymer composites in lightweight automotive applications. Analysis of these fibers, averaging 35 mm in length and 300 µm in diameter, revealed cellulose as their primary component and relatively low lignin content. To enhance their strength and reinforcing potential, the fibers underwent chemical modification via an alkali treatment.

Theoretical models, which considered the microfibrillar angle, predicted a strength range of 192.8 to 238.5 MPa. However, experimental results yielded significantly lower values, ranging between 112.5 and 147.5 MPa. This disparity suggests that the predicted strengths exceeded the experimental findings, possibly due to the fiber’s intrinsic cellular structure or the empirical models used. By applying an adjusted empirical Eq. (7) to fibers with an average length of 35 mm and a diameter of 300 µm, strength values between 113.25 and 140.69 MPa were derived, closely aligning with the experimental data. Further morphological examination clarified the fiber’s structural behavior.

In summary, this comprehensive study —encompassing the chemical composition, surface modification, morphology, and physical and mechanical properties of areca fibers—demonstrates their significant potential for developing sustainable, high-performance materials. Areca fiber treatments are generally more cost-effective than those for other natural fibers like jute or coir. Its abundance and biodegradability further position areca fiber as a cost-effective choice for enhancing composite properties. The primary expenses associated with areca fiber treatment include the cost of the fiber itself, along with treatment chemicals (e.g., alkali) or equipment for physical processing. However, methods such as hammer milling and cyclone separation can damage areca fibers, potentially compromising their mechanical properties and suitability for specific applications.

Ultimately, this research could lead to enhanced composites with improved strength, durability, and biodegradability, providing a viable alternative to conventional synthetic materials across various industries. Integrating treated areca fibers into specialized material applications, particularly for additive manufacturing (3D printing) filaments, introduces specific formulation obstacles. Achieving a uniform dispersion of fibers remains a persistent challenge; agglomeration of fibers often leads to inconsistencies in filament diameter, frequently resulting in extrusion failures and blockages of the printing nozzle. The commercial scaling of fiber surface modification also faces dual hurdles: the inherent variability in raw material quality inevitably leads to non-uniform end products, and the environmental expenses associated with the management of chemical effluents from these processes are often prohibitive. However, the physiochemical characteristics and the performance of surface treated areca fibers reported in this study are limited to the raw material extracted from Karnataka region of India and the processing conditions. Predictive methods like machine learning and computational modeling will play a major role in future to identify optimal process parameters, eliminating the need for costly physical testing and material waste.

## Data Availability

The datasets used and/or analysed during the current study available from the corresponding author (nr_banapurmath@kletech.ac.in) on a reasonable request.

## References

[1] Palanisamy, S. et al. Characterization of *Acacia caesia* bark fibers (ACBFs). *J. Nat. Fibers* **19**(15), 10241–10252. 10.1080/15440478.2021.1993493 (2021).

[2] Almeshaal, M., Palanisamy, S., Murugesan, T. M., Palaniappan, M. & Santulli, C. Physico-chemical characterization of Grewia Monticola Sond (GMS) fibers for prospective application in biocomposites. *J. Nat. Fibers* **19**(17), 15276–15290. 10.1080/15440478.2022.2123076 (2022).

[3] Palaniappan, M. et al. Novel *Ficus retusa* L. aerial root fiber: a sustainable alternative for synthetic fibres in polymer composites reinforcement. *Biomass Conv. Bioref.* **15**, 7585–7601. 10.1007/s13399-024-05495-4 (2025).

[4] Palanisamy, S. et al. Tailoring epoxy composites with *Acacia caesia* bark fibers: evaluating the effects of fiber amount and length on material characteristics. *Fibers* **11**, 63. 10.3390/fib11070063 (2023).

[5] Palanisamy, S., Kalimuthu, M., Nagarajan, R., Fernandes Marlet, J. M. & Santulli, C. Physical, chemical, and mechanical characterization of natural bark fibers (NBFs) reinforced polymer composites: a bibliographic review. *Fibers* **11**, 13. 10.3390/fib11020013 (2023).

[6] Ayrilmis, N., Kanat, G., Yildiz Avsar, E., Palanisamy, S. & Ashori, A. Utilizing waste manhole covers and fibreboard as reinforcing fillers for thermoplastic composites. *J. Reinforced Plastics Compos.*10.1177/07316844241238507 (2024).

[7] Suresh, T., Elango, M., Venkatachalam, G. & Shenbaga, V. P. Areca fiber reinforced bio-materials: a review on processing, properties and advanced optimization technique. *J. Nat. Fibers* **21**(1), 2357236. 10.1080/15440478.2024.2357236 (2024).

[8] Gokarneshan, N., Sathya, V., Lavanya, J., Shabnum, S. & Anton, S. M. A Review of significant advances in areca fiber composites. *Next Gener. Text.*10.5772/intechopen.108028 (2022).

[9] Food and Agriculture Organization of the United Nations, “FAOSTAT”, Food and Agriculture Data. http://faostat.fao.org/. (2014).

[10] Gokarneshan, N., Sathya, V., Lavanya, J. & Shabnum, S. A review of significant advances in Areca fiber composites. Next-generation textiles. *IntechOpen*10.5772/intechopen.108028 (2023).

[11] Ali Mekouar, M. Food and agriculture organization of the United Nations (FAO). *Yearbook Int. Environ. Law* **28**, 506–520. 10.1093/yiel/yvy073 (2017).

[12] Binoj, J. S., Raj, R. E., Sreenivasan, V. S. & Thusnavis, G. R. Morphological, physical, mechanical, chemical and thermal characterization of sustainable Indian areca fruit husk fibers (*Areca Catechu* L.) as potential alternate for hazardous synthetic fibers. *J. Bionic Eng.* **13**(1), 156–165. 10.1016/S1672-6529(14)60170-0 (2016).

[13] Loganathan, T. M. et al. Physical, thermal and mechanical properties of areca fiber reinforced polymer composites — an overview. *J. Bionic Eng.* **17**(1), 185–205. 10.1007/s42235-020-0015-6 (2020).

[14] Shenoy, H. S. et al. Comparative evaluation of chemical treatment on the physical and mechanical properties of areca frond, banana, and flax fibers. *J. Nat. Fibers* **19**(4), 1531–1543. 10.1080/15440478.2020.1784817 (2022).

[15] Jothibasu, S., Mohanamurugan, S. & Vinod, A. Influence of chemical treatments on the mechanical characteristics of areca sheathflax fibres based epoxy composites. *Rasayan J. Chem.* **11**(3), 1255–1262. 10.31788/RJC.2018.1133096 (2018).

[16] Dhanalakshmi, S., Ramadevi, P. & Basavaraju, B. A study of the effect of chemical treatments on areca fiber reinforced polypropylene composite properties. *Sci. Eng. Compos. Mater.* **24**(4), 501–520. 10.1515/secm-2015-0292 (2017).

[17] Uvaraja, V. C., Sureshkumar, K., Radha Krishnan, B., Saravanan, G. & Ramakrishnan, H. Characterization of untreated and alkali treated Areca catechu fibre through eco-friendly composite plates. *Mater. Today Proc.*10.1016/j.matpr.2021.11.004 (2022).

[18] Nayak, S. & Mohanty, J. R. Influence of chemical treatment on tensile strength, water absorption, surface morphology, and thermal analysis of areca sheath fibers. *J. Nat. Fibers* **16**(4), 589–599. 10.1080/15440478.2018.1430650 (2019).

[19] Ashok, R. B., Srinivasa, C. V., Sakshi, S. K. & Basavaraju, B. Tensile and flexural properties of areca sheath fibers. *Mater. Today Proc.* **5**, 28080–28088. 10.1016/j.matpr.2018.10.049 (2018).

[20] Kayambu, A. & Ramasubbu, R. Physical chemical and surface morphological characterization of areca catechu fiber. *J. Nat. Fibers* **19**(15), 11435–11448. 10.1080/15440478.2022.2025981 (2022).

[21] Kencanawati, C. I. P. K., Suardana, N. P. G., Sugita, I. K. G. & Suyasa, I. W. B. A study on biocomposite from local Balinese *Areca catechu* L. husk fibers as reinforced material. *IOP Conf. Ser. Mater. Sci. Eng.*10.1088/1757-899X/201/1/012002 (2017).

[22] Ramasubbu, R., Kayambu, A., Palanisamy, S. & Ayrilmis, N. Mechanical properties of epoxy composites reinforced with *Areca catechu* fibers containing silicon carbide. *BioResources* **19**(2), 2353–2370 (2024).

[23] Suresh, P. S., Dilip, K. K., Dhanalakshmi, S. & Basavaraju, B. Physical, chemical and surface morphological characterization of single areca sheath fiber. *IOP Conf. Ser. Mater. Sci. Eng.*10.1088/1757-899X/1065/1/012020 (2021).

[24] Suresh, P. S., Dilip, K. K., Basavaraju, B. & Atul, B. Plain-woven areca sheath fiber-reinforced epoxy composites: the influence of the fiber fraction on physical and mechanical features and responses of the tribo system and machine learning modeling. *ACS Omega* **9**(7), 8019–8036. 10.1021/acsomega.3c08164 (2024). 38405460 10.1021/acsomega.3c08164 PMC10882675

[25] Sakshi, S. K. & Basavaraju, B. Enhanced mechanical properties of potassium permanganate treated areca sheath fibre epoxy composite. *Mater. Today Proc.* **66**, 2274–2281. 10.1016/j.matpr.2022.06.222 (2022).

[26] Kamath, S. S. et al. Tensile and flexural behaviour of areca husk fibre reinforced epoxy composite. In *Advances in Metrology and Measurement of Engineering Surfaces Lecture Notes in Mechanical Engineering* (eds Prakash, C. et al.) (Springer, 2021).

[27] Sakshi, S. K., Sunil, B. & Basavaraju, B. Tribological studies of epoxy composites using surface modified areca sheath fibres. *Mater. Today Proc.*10.1016/j.matpr.2021.01.193 (2021).

[28] Banagar, A., Chikkol, S. V. & Bennehalli, B. Studies on physical and mechanical properties of untreated (raw) and treated areca leaf sheaths. *Mater. Res. Innovations* **25**(7), 404–411. 10.1080/14328917.2020.1834747 (2020).

[29] Sakshi, S. K. & Basavaraju, B. Potential of using areca fibres in composite fabrication. *Mater. Today Proc.* **44**, 4143–4149. 10.1016/j.matpr.2020.10.461 (2021).

[30] Suresh, P. S., Dilip Kumar, K., Dhanalakshmi, S., Srinivasa, C. V. & Basavaraju, B. Effect of fiber fraction on the physical and mechanical properties of short areca sheath fiber reinforced polymer composite. *Mater. Today Proc.*10.1016/j.matpr.2020.12.892 (2021).

[31] Ashok, R. B., Srinivasa, C. V. & Basavaraju, B. Study on morphology and mechanical behavior of areca leaf sheath reinforced epoxy composites. *Adv. Compos. Hybrid Mater.* **3**, 365–374. 10.1007/s42114-020-00169-x (2020).

[32] Binoj, J. S. et al. Taguchi’s optimization of areca fruit husk fiber mechanical properties for polymer composite applications. *Fibers Polym.* **23**, 3207–3213. 10.1007/s12221-022-0365-2 (2022).

[33] Ramasubbu, R., Kayambu, A., Palanisamy, S. & Ayrilmis, N. Mechanical properties of epoxy composites reinforced with *Areca catechu* fibers containing silicon carbide. *BioResources* **19**(2), 2353–2370. 10.15376/biores.19.2.2353-2370 (2024).

[34] Pan, Y. et al. Effect of silane coupling agent on modification of areca fiber/natural latex. *Materials*10.3390/ma13214896 (2020). 33142806 10.3390/ma13214896 PMC7663144

[35] Vishnu, K. S., Anuroop, P. A., Anto, L. P., Mathew, L. & Shunmugesh, K. Areca fiber reinforced LLDPE biocomposite. *Mater. Today Proc.* **24**, 1924–1931. 10.1016/j.matpr.2020.03.619 (2019).

[36] Shanmugasundaram, N., Rajendran, I. & Ramkumar, T. Characterization of untreated and alkali treated new cellulosic fiber from an Areca palm leaf stalk as potential reinforcement in polymer composites. *Carbohyd. Polym.* **195**, 566–575. 10.1016/j.carbpol.2018.04.127 (2018). 10.1016/j.carbpol.2018.04.127 29805013

[37] Deng, T., Liu, Y., Huang, X.-N. & Lin, W.-M. Raw material analysis and pulping properties study of areca-nut. *Chung-kuo Tsao Chih China Pulp Paper* **36**(9), 39–42. 10.11980/j.issn.0254-508X.2017.09.008 (2017).

[38] Balreddy, M. S. et al. Utilization of alkali-treated areca fibers for stabilizing silty sand soil for use in pavement subgrades: Analysis using IITPAVE software. *Emergent Mater.* **7**, 1927–1939. 10.1007/s42247-024-00710-4 (2024).

[39] Sunny, G. & Rajan, T. P. Review on areca nut fiber and its implementation in sustainable products development. *J. Nat. Fibers* **19**(12), 4747–4760. 10.1080/15440478.2020.1870623 (2021).

[40] Gokarneshan, N. et al. A review of significant advances in areca fiber composites. Next-generation textiles. *IntechOpen*10.5772/intechopen.108028 (2023).

[41] Raghu, P. G. & Ranganagowda, S. S. Extraction and characterization of cellulose from natural areca fiber. *Mater. Sci. Res. India* **16**(1), 86–93. 10.13005/msri/160112 (2019).

[42] Arumugam, K. & Ramasubbu, R. Physical chemical and surface morphological characterization of *Areca catechu* fiber. *J. Nat. Fibers* **19**(15), 1–14. 10.1080/15440478.2022.2025981 (2022).

[43] Poornima, H. L., Muralidhar, N. & Praveen, J. V. Mechanical characterization of areca fine fiber fabric (AFFF) reinforced epoxy composites. *Mater. Today Proc.*10.1016/j.matpr.2022.05.589 (2022).

[44] Roopashree, C. R. & Shivarudraiha, H. K. S. The study on the mechanical properties of areca nut: A review. *Mater. Today Proc.*10.1016/j.matpr.2023.04.492 (2023).

[45] Vasudeva Nayaka, K. B. L. & Rangaswamy, B. E. Areca husk fibers as agro-waste to value added products in textile sector - A practicability study. *Int. J. Adv. Res.* **7**(9), 106–111 (2019).

[46] Desai, R. H., Krishnamurthy, L. & Shridhar, T. N. Effectiveness of Areca (Betel) fiber as a reinforcing material in eco-friendly composites: a review. *Indian J. Adv. Chem. Sci.* **S1**, 27–33 (2016).

[47] Dinakaran, K., Ramesh, H., Joseph, A. D., Murugan, R. & Jothi, S. Development and characterization of areca fiber reinforced polymer composite. *Mater. Today Proc.*10.1016/j.matpr.2019.06.528 (2019).

[48] Naik, P., Kumar, V., Sunil, K. S. & Srinivasa, K. R. A Study of short areca fiber and wood powder reinforced phenol formaldehyde composites. *Am. J. Mater. Sci.*10.5923/c.materials.201502.28 (2015).

[49] Ashok, R. B., Srinivasa, C. V. & Basavaraju, B. A review on the mechanical properties of areca fiber reinforced composites. *Sci. Technol. Mater.* **30**(2), 120–130. 10.1016/j.stmat.2018.05.004 (2018).

[50] Georgy Sunny, T. & Rajan, P. An effect of retting process and extraction methods on physical and mechanical properties of areca nut fibers. *Int. J. Cloth. Sci. Technol.* **36**(4), 722–739. 10.1108/IJCST-09-2023-0133 (2024).

[51] Lazim, Y., Salit, S. M., Zainudin, E. S., Mustapha, M. & Jawaid, M. Effect of alkali treatment on the physical, mechanical, and morphological properties of waste betel nut (*Areca catechu*) husk fibre. *BioRes* **9**(4), 7721–7736 (2014).

[52] Makruf, Z. I., Afnison, W. & Rahim, B. A Study on the utilization of areca nut husk fiber as a natural fibre reinforcement in composite applications: A systematic literature review. *J. Eng. Res. Lect.* **3**(1), 18–28. 10.58712/jerel.v3i1.123 (2024).

[53] Srinivasa, C. V. & Bharath, K. N. Effect of alkali treatment on impact behavior of areca fibers reinforced polymer composites. *Int. J. Mater. Eng.* **7**(4), 875–879 (2013).

[54] Jeevan Rao, H. et al. Effect of chemical treatment on physio-mechanical properties of lignocellulose natural fiber extracted from the bark of careya arborea tree. *Heliyon* **10**(5), e26706. 10.1016/j.heliyon.2024.e26706 (2024). 38434283 10.1016/j.heliyon.2024.e26706 PMC10907790

[55] Hariharashayee, D. Experimental investigation of raw and alkali treated areca leaf stalk and sisal fibres reinforced hybrid polymer composites. *Wutan Huatan Jisuan Jishu* **16**, 273–283 (2020).

[56] Rangaraj, R. et al. Investigation of weight fraction and alkaline treatment on *Catechu Linnaeus/Hibiscus cannabinus/Sansevieria Ehrenbergii* plant fibers-reinforced epoxy hybrid composites. *Adv. Mater. Sci. Eng.*10.1155/2022/4940531 (2022).

[57] Mahmud, R. U., Islam, M. S., Hossain, S., Nasrin, J. & Khan, A. N. Agro-waste areca nut husk and bagasse fiber reinforced epoxy-based hybrid composite for thermal insulated false ceiling application. *SPE Polym.* **6**(1), e10166. 10.1002/pls2.10166 (2025).

[58] Hindi, J., Muralishwara, K. & Gurumurthy, B. M. Comparative analysis of physical, morphological, tensile and thermal stability characteristics of raw and alkali treated novel *Tinospora cordifolia* natural fiber. *Sci. Rep.* **15**(1), 18596. 10.1038/s41598-025-03627-y (2025). 40425676 10.1038/s41598-025-03627-y PMC12116738

[59] Dhanalakshmi, S., Basavaraju, B. & Ramadevi, P. Areca fiber reinforced epoxy composites: effect of chemical treatments on impact strength. *Orient J. Chem.* **31**(2), 763–769 (2015).

[60] Binoj, J. S., Jaafar, M., Mansingh, B. & Karu, C. V. Extensive characterization of pretreated/enzyme treated cellulosic *Areca catechu* L. peduncle fiber for polymer composite applications. *J. Appl. Polym. Sci.* **140**(32), e54248. 10.1002/app.54248 (2023).

[61] Mukherjee, P. S. & Satyanarayana, K. G. Structure and properties of some vegetable fibres. *J. Mater. Sci.* **19**, 3925–3934. 10.1007/BF00980755 (1984).

[62] Mohanakumar G.C. A study of short areca fiber reinforced PF composites. *Proceeding of the World Congress on Engineering 2008 (WCE 2008) held at London, U.K. during July 2–4*, (2008).

[63] Swamy, R. P., Kumar, G. C. M., Vrushabhendrappa, Y. & Joseph, V. Study of areca-reinforced phenol formaldehyde composites. *J. Reinf. Plast. Compos.* **23**(13), 1373–1382. 10.1177/0731684404037049 (2004).

[64] Rosa, M. F. et al. Effect of fiber treatments on tensile and thermal properties of starch/ethylene vinyl alcohol copolymers/coir biocomposites. *Bioresour. Technol.* **100**(21), 5196–5202. 10.1016/j.biortech.2009.03.085 (2009). 19560341 10.1016/j.biortech.2009.03.085

[65] Ramli, R., Shaler, S. M. & Jamaludin, M. A. Properties of medium density fibreboard from oil palm empty fruit bunch fibre. *J. Oil Palm Res.* **14**, 34–40 (2002).

[66] Manavendra, G., Kumarappa, S., Baskar Dixit, C. S. & Mohan Kumar, G. C. Characterization of areca fiber reinforced phenol formaldehyde composites. *Int. J. Mater. Sci.* **5**(3), 299–308 (2010).

[67] Sreekala, M. S., Kumaran, M. G. & Thomas, S. Oil palm fibers: morphology, chemical composition, surface modification, and mechanical properties. *J. Appl. Polym. Sci.* **66**(5), 821–835 (1997).

[68] Rekha, B. & NagarajaGanesh, B. X-ray diffraction: an efficient method to determine microfibrillar angle of dry and matured cellulosic fibers. *J. Nat. Fibers* **19**(10), 3689–3696. 10.1080/15440478.2020.1848720 (2020).

[69] NagarajaGanesh, B. & Rekha, B. Intrinsic cellulosic fiber architecture and their effect on the mechanical properties of hybrid composites. *Archiv. Civ. Mech. Eng* **20**, 125. 10.1007/s43452-020-00125-y (2020).

[70] NagarajaGanesh, B. & Rekha, B. Effect of mercerization on the physico-chemical properties of matured and seasoned *Cocos nucifera* fibers for making sustainable composites. *Mater. Res. Express*10.1088/2053-1591/ab5395 (2019).

[71] Dhanalakshmi, S., Ramadevi, P., Basavaraju, B., Raghu, P. R. & Srinivasa, C. V. Natural areca fiber: surface modification and spectral studies. *J. Adv. Chem.* **10**(10), 3263–3273 (2014).

[72] Raghu Patel, G. R., Sakshi, S. K. & Basavaraju, B. Extraction and characterization of cellulose from natural areca fiber. *Mater. Sci. Res. India* **16**(1), 86–93. 10.13005/msri/160112 (2019).

[73] Palanisamy, S., Ramakrishnan, S. K., Santulli, C., Khan, T. & Ahmed, O. S. Mechanical and wear performance evaluation of natural fiber/epoxy matrix composites. *BioResources* **19**(4), 8459–8478 (2024).

[74] Sireesha Koneru, A., Srinath, B. N. & Rao, T. B. Simplified optimal design of NU202 cylindrical roller bearing and validation through GA. *Int. J. Interact. Design Manuf. (IJIDeM)*10.1007/s12008-023-01402-9 (2023).

[75] Koneru, S. et al. Optimal lapping and polishing process parameters for finer surface finishing of GCr15 steel cylindrical roller bearings. *Int. J. Interact. Des. Manuf.*10.1007/s12008-023-01555-7 (2023).

[76] Dharmendra, B. V., Kodali, S. P. & Nageswara Rao, B. A simple and reliable Taguchi approach for multi-objective optimization to identify optimal process parameters in nano-powder-mixed electrical discharge machining of INCONEL800 with copper electrode. *Heliyon* **5**, e02326. 10.1016/j.heliyon.2019.e02326 (2019). 31485524 10.1016/j.heliyon.2019.e02326 PMC6716227

[77] Anantha, M. T. et al. Surface quality improvement of AZ31 Mg alloy by a combination of modified Taguchi method and simple multi objective optimization procedure. *Cogent Eng.* **11**(1), 2338476. 10.1080/23311916.2024.2338476 (2024).

[78] Reddy, K. P. K., Ramesh Kumar, S. & Rao, B. N. Multi-objective optimization for minimize cutting force and power consumption in corn stalk chopping processes. *Heliyon* **10**, e35373. 10.1016/j.heliyon.2024.e35373 (2024). 39165957 10.1016/j.heliyon.2024.e35373 PMC11334816

